# Balancing Data Quality and Bias: Investigating Functional Connectivity Exclusions in the Adolescent Brain Cognitive Development℠ (ABCD Study) Across Quality Control Pathways

**DOI:** 10.1002/hbm.70094

**Published:** 2025-01-09

**Authors:** Matthew Peverill, Justin D. Russell, Taylor J. Keding, Hailey M. Rich, Max A. Halvorson, Kevin M. King, Rasmus M. Birn, Ryan J. Herringa

**Affiliations:** ^1^ Department of Psychiatry University of Wisconsin–Madison Madison, WI USA; ^2^ Department of Psychology Yale University New Haven, CT USA; ^3^ School of Social Work University of Washington Seattle, WA USA; ^4^ Department of Psychology University of Washington Seattle, WA USA

**Keywords:** ABCD, adolescents, missing data, motion, quality control, rs‐fMRI

## Abstract

Analysis of resting state fMRI (rs‐fMRI) typically excludes images substantially degraded by subject motion. However, data quality, including degree of motion, relates to a broad set of participant characteristics, particularly in pediatric neuroimaging. Consequently, when planning quality control (QC) procedures researchers must balance data quality concerns against the possibility of biasing results by eliminating data. In order to explore how researcher QC decisions might bias rs‐fMRI findings and inform future research design, we investigated how a broad spectrum of participant characteristics in the Adolescent Brain and Cognitive Development (ABCD) study were related to participant inclusion/exclusion across versions of the dataset (the ABCD Community Collection and ABCD Release 4) and QC choices (specifically, motion scrubbing thresholds). Across all these conditions, we found that the odds of a participant's exclusion related to a broad spectrum of behavioral, demographic, and health‐related variables, with the consequence that rs‐fMRI analyses using these variables are likely to produce biased results. Consequently, we recommend that missing data be formally accounted for when analyzing rs‐fMRI data and interpreting results. Our findings demonstrate the urgent need for better data acquisition and analysis techniques which minimize the impact of motion on data quality. Additionally, we strongly recommend including detailed information about quality control in open datasets such as ABCD.

## Graphical abstract

This study investigated participant characteristics associated with exclusion of resting state fMRI data across a range of quality control procedures. Many participant characteristics made exclusion more likely across conditions, demonstrating that correction for missing data is necessary for accurate estimates of brain‐behavior relationships when examining functional connectivity.

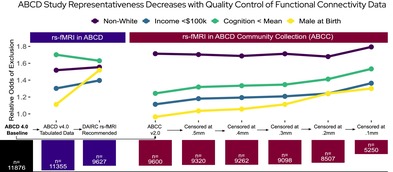

**Summary**

- Excluding participants corrects for quality artifacts in functional connectivity data but this list‐wise deletion also biases statistics because exclusions are not completely at random.
- Biases resulting from exclusions are likely even with relatively liberal standards for acceptable data quality.
- Missing data handling strategies such as multiple imputation should be used to correct biases from non‐random exclusions in functional connectivity analyses.

Resting‐state functional magnetic resonance imaging (rs‐fMRI) data are inherently noisy and distorted by artifacts—particularly from participant motion (Power et al. 2012). Poor quality images are typically excluded from analysis during quality control (QC) to control for artifacts. These exclusion decisions are often subject both to researchers' subjective judgments (e.g., following visual inspection) and variability in research practices (e.g., a numeric threshold for allowable motion; Taylor et al. 2023). Importantly, participant‐level characteristics that may be central to research questions can be potent predictors of motion during scan, in turn influencing data quality and exclusion from analysis (Cosgrove et al. 2022; Hausman et al. 2022; Hodgson et al. 2017; Satterthwaite et al. 2012). The practice of removing an entire case (i.e., a participant) from a dataset when a portion of the data for that case is missing is known as listwise deletion—a practice known to bias results, particularly when study variables correlate with the probability of missingness (Baraldi and Enders 2010; Woods et al. 2023). Researchers must therefore balance competing desires to maximize statistical power, minimize risk of spurious findings, and report unbiased results. However, there is little empirical guidance available to inform such an effort. The current manuscript uses rs‐fMRI data from the Adolescent Brain Cognitive Development (ABCD) Study to investigate relations between a variety of participant characteristics with odds of exclusion according to a range of typical QC practices.

Participants, especially children, are most commonly excluded from rs‐fMRI studies due to excess in‐scanner head motion (Frew et al. 2022; Satterthwaite et al. 2012). Head motion during a scan produces motion‐related signal in areas of the brain which are moving together (e.g., between regions of interested located at the anterior and posterior poles of the brain during a nodding motion). This signal is a confound in analyses examining the relation of motion‐related participant characteristics, such as age, with functional connectivity (Power et al. 2012; Power, Schlaggar, and Petersen 2015). Importantly, in‐scanner motion can be expected to relate to the broad spectrum of neurodevelopmental, demographic, and environmental factors related to variability in the development of inhibitory control in childhood (McNeilly et al. 2021; Noble, McCandliss, and Farah 2007; Rhoades et al. 2011), and previous work has confirmed associations between motion and participant characteristics including demographic factors, executive functioning, psychopathology, and body mass index (BMI; Cosgrove et al. 2022; Gard et al. 2023; Kay et al. 2023; Satterthwaite et al. 2012).

A variety of methods exist for controlling motion‐related signal. Without removing data, analysts can statistically control for observed motion, control for signal originating outside of neural tissue (aCompCor; Behzadi et al. 2007), or use independent component analysis (ICA) to attempt to remove motion‐related signal (Pruim et al. 2015). Other techniques involve censoring of high motion frames from analysis via scrubbing (Power et al. 2014) or spike regression (Ciric et al. 2018; Satterthwaite et al. 2013). Volume censoring results in the exclusion of participants who lack sufficient data for reliable connectivity estimation following censoring. However, with the possible exception of ICA, non‐censoring methods do not appear to control for noise artifact as effectively as censoring methods unless high motion participants are otherwise excluded from the data (Parkes et al. 2018). For this reason, rs‐fMRI analyses typically exclude a sizeable proportion of the original sample. Importantly, because exclusion is contingent on motion, which is related to other participant characteristics, the missingness introduced by this censoring will not be completely random (Baraldi and Enders 2010).

While the consequences of including poor quality data are clear, the negative consequences of list‐wise deletion of rs‐fMRI data have garnered less attention. First, eliminating data may exacerbate concerns over small sample sizes in neuroscience (Marek et al. 2022), particularly when studying difficult to recruit and/or potentially high‐motion participants such as children with ADHD. Second, listwise deletion of participant data in the presence of associations between study variables (such as disinhibition) and exclusion will bias statistical estimates (Baraldi and Enders 2010). Third and finally, regardless of the research question, QC‐related exclusions may limit generalizability of findings should the factors driving exclusion vary across populations, contributing to a chronic lack of population‐representative samples in cognitive neuroscience research (Dotson and Duarte 2020; Fakkel et al. 2020; Green et al. 2022). Conversely, reports on findings drawn from poor quality data gathered from underrepresented subpopulations could introduce a countervailing bias and distorted findings, necessitating thoughtful consideration of QC approaches.

In short, researchers must carefully balance concerns about data quality against the consequences of removing data from analysis, and there is a lack of empirical guidance on how to best do so. This ambiguity opens the door for the dissemination of biased findings and increases researcher degrees of freedom—which may lead to higher rates of false positives in the literature (Gelman and Loken 2013). Large neuroimaging datasets such as the ABCD study provide an ideal opportunity to systematically explore the impact of various QC decisions on both data quality and representativeness—indeed, the use of the ABCD Study's own recommendations regarding rs‐fMRI inclusion is known to result in a sample that does not fully represent American youth (Cosgrove et al. 2022). Critically, the broader impacts of various researcher decision‐points regarding QC (e.g., motion thresholding) remain poorly understood in the context of the ABCD Study or most other “big data” researcher initiatives. Here, we explore the relationships between 16 ABCD participant characteristics at baseline with exclusion according to a range of research practices (including choice of motion threshold), as well as information about data quality at every level of exclusion. We hypothesized that each variable would be related to missingness in at least some conditions, and that these biases would become larger as more data was excluded by QC procedures.

## 1 Materials and Methods

The ABCD project is designed to follow a sample of 11,836 nine‐ and 10‐year‐olds over 10 years of development, collecting lab‐based assessments annually and neuroimaging data biennially. Baseline data collection was completed in 2018. Participants were recruited at 21 sites across the United States. For inclusion, participants had to be between 9 and 10 years of age, and able to provide informed consent (parents) and assent (children). Exclusion criteria included lack of English language proficiency (child), severe sensory intellectual, medical, or neurological issues that would impact the validity of data, inability to comply with the protocol, and contraindications for MRI (Thompson et al. 2019). While site selection was constrained by institutional resources, a pseudo‐representative sample was recruited by employing probability sampling within the combined catchment area which encompassed 20% of US nine‐ and 10‐year‐olds (Garavan et al. 2018). To aid genetic analysis, twins were over‐sampled (Iacono et al. 2018). Only baseline data was analyzed in this study.

Behavioral and demographic variables, as well as imaging QC variables including scanner type, acquisition site, and recommended inclusion flags from the ABCD Data Analysis, Informatics & Resource Center (DAIRC) were downloaded as part of ABCD Data Release v4.0 (Jernigan et al. 2021). Methods and analyses for the present study were preregistered (https://osf.io/57xer/), with minor deviations described in our Supporting Information. Informed consent was obtained from all ABCD participants and their parents during data collection; data collection was approved by the IRBs of the University of California, San Diego, as well as IRBs at each data collection site (D. B. Clark et al. 2018; Garavan et al. 2018).

### 1.1 Behavioral and Demographic Measure

A wide range of behavioral and demographic measures were examined for systematic relation with exclusion (see Table 1). These were chosen based either on known relation to functional connectivity or motion and/or their importance as metrics of sample representativeness. Neighborhood factors included Area Deprivation Index (ADI; Singh 2003) and Child Opportunity Index v2.0 (COI; Fan et al. 2021). Trauma exposure was categorized as unexposed, single exposure, or multiple (two or more) exposures using the parent‐report Kiddie Schedule for Affective Disorders and Schizophrenia (KSADS; Townsend et al. 2020). Total family income and highest parental education were collected using the ABCD demographic questionnaire and re‐leveled to allow for larger subject counts within each level (see Heeringa and Berglund 2020). We used U.S. Census derived race/ethnicity categories (“Black”, “Hispanic”, “Asian”, “White”, or “Other”), included in ABCD to facilitate comparisons with existing literature (e.g., Cosgrove et al. 2022; Fadus et al. 2021; Heeringa and Berglund 2020); notably, however, this approach may under‐represent important U.S. sub‐populations (Saragosa‐Harris et al. 2022). Secondary analyses using more detailed demographics are included in Supporting Information. Pubertal status was included as measured by the Pubertal Development Scale (PDS; Petersen et al. 1988). In order to separately examine the impact of general, internalizing, and externalizing psychopathology, we calculated a generalized psychopathology factor (P), as well as residual internalizing and externalizing factors, by fitting a bifactor model to the 8 subscales of the Child Behavior Checklist (CBCL; Achenbach, Dumenci, and Rescorla 2002; Brislin et al. 2021; D. A. Clark et al. 2021). Cognitive ability was operationalized using the matrix reasoning task scaled score from the WISC‐V as well as the cognition composite and crystallized intelligence composite scores captured by the NIH Toolbox (Akshoomoff et al. 2013; Thompson et al. 2019). The NIH Toolbox Flanker Inhibitory Control task score (Weintraub et al. 2013) was included as a behavioral measure of inhibitory control. Body Mass Index was calculated using measured height and weights and standardized, with adjustment for participant age and sex, using WHO norms (Myatt and Guevarra 2019). Further information about measure selection and validity is presented in the Supporting Information.

**TABLE 1:** Variable descriptions.

Construct	Measures
Resting state connectivity	Correlation (connectivity) matrices derived from the Glasser et al. (2016) parcellation.
Image quality/inclusion	Inclusion in ABCC Inclusion in ABCD release 4.0 Tabulated Data Recommended for rsfMRI analysis by DAIRC
Average in‐scanner head motion	Average framewise displacement during the rs‐fMRI scan
Neighborhood Disadvantage (see Fan et al. 2021)	Area disadvantage index Child opportunity index V2
Trauma/stress exposure	K‐SADS PTSD Criterion‐A exposure count
Parent reported participant demographics	Age Sex assigned at birth Race/ethnicity (census categories) Total household income Highest parental education
Pubertal development	Pubertal development scale derived category scores (Cheng et al. 2021)
Psychopathology (p, INT, EXT)	Orthogonal factors calculated from child behavior checklist
General cognitive ability	WISC‐V matrix reasoning scaled score NIH‐Toolbox crystallized intelligence composite score NIH‐Toolbox cognition composite score
Inhibitory control	NIH toolbox flanker inhibitory control task
Body mass index	Age‐ and sex‐corrected Z‐scored BMI

*Note:* Additional measurement detail is included in the Supporting Information.

### 1.2 Imaging Measures

MRI images were collected using 3 T scanners by multiple manufacturers across the acquisition sites. Resting state fMRI was collected in 4, five‐minute multi‐slice/multiband EPI scans, acquired axially (matrix = 90 × 90; 60 slices; FOV = 216 × 216 mm; resolution = 2.4 × 2.4 × 2.4 mm; TR = 800 ms; TE = 30 ms; flip angle = 52°; MultiBand acceleration factor = 6); during acquisition, participants rested with eyes open and passively viewed a cross‐hair image (Casey et al. 2018). All rs‐fMRI Data (including motion estimates) for this analysis were obtained from the ABCD Community Collection (ABCC; data release 2.0.0). Pre‐processing by ABCC included segmentation of structural images, registration to a template surface, projection of functional images to the template surface, demeaning and detrending resting state timeseries with respect to time, motion estimation (including filtering of factitious motion secondary to magnetic field changes caused by breathing), denoising (regression of white matter, CSF, mean timeseries signal, and motion parameters), and bandpass filtering between 0.008 and 0.09 Hz (Feczko et al. 2021). Functional connectivity matrices were estimated from regions‐of‐interest (*k* = 180) defined in the parcellation developed by Glasser et al. (2016).

### 1.3 Inclusion/Exclusion Flags

**FIGURE 1:**
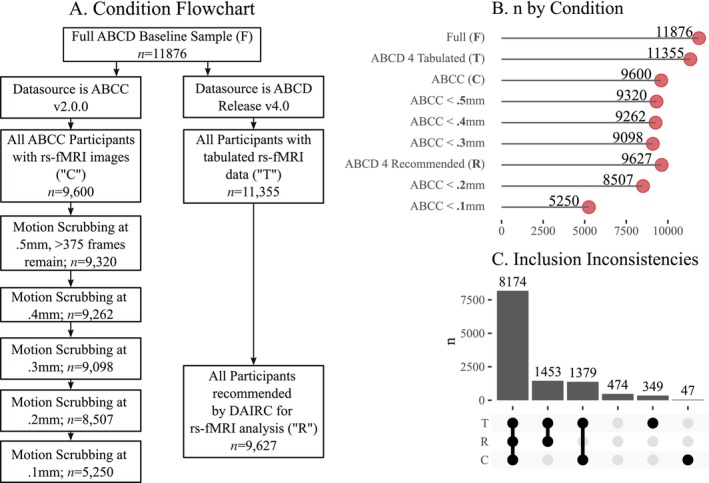
9 samples were generated based on quality control conditions. (A) shows the path diagram leading to the various conditions. (B) Shows n by Condition. (C) Illustrates non‐overlap between the ABCD Tabulated, ABCD Recommended, and ABCC conditions.

Eight recommended inclusion/exclusion flags were calculated based on variables provided by two processing streams: ABCC and ABCD (see Figure 1). These were designed to simulate eight approaches to the data that potential investigators might choose, starting with the choice of processing stream.

#### 1.3.1 ABCC Conditions

Images and information in the ABCC repository originally derive from ABCD “Fast‐track” (unprocessed; https://abcdstudy.org/scientists/data‐sharing/fast‐track‐imaging‐data‐release/) imaging data. Participants with required scans denoted as “useable” quality in the attached operator QC reports were considered for inclusion by the ABCC investigator team. Our ‘ABCC’ condition included all participants in this repository with pre‐processed (though not motion‐corrected) rs‐fMRI data (Feczko et al. 2021).

Data from participants in the ABCC condition was subsequently winnowed into five motion thresholding conditions corresponding to thresholds of 0.5, 0.4, 0.3, 0.2, and 0.1 mm. These conditions were constructed from frame censoring masks, included in ABCC, which flagged frames for exclusion which either showed motion (framewise displacement, or FD) exceeding the threshold or which occurred in an interval of no more than five contiguous non‐censored frames between two such high‐motion frames (Power et al. 2014). Respiratory artifact was filtered from FD values prior to scrubbing (Fair et al. 2020). Participants with fewer than 375 frames (5 min) of un‐scrubbed data following scrubbing using a given threshold were considered excluded in that condition. This threshold is common in neuroimaging (Finn et al. 2015; Power et al. 2014; Power, Schlaggar, and Petersen 2015; Van Dijk et al. 2010), and consistent with DAIRC's ABCD release recommendations (including those in release v4.0).

#### 1.3.2 ABCD Release v4.0 Conditions

We separately examined two conditions from the DAIRC pre‐processed imaging data from ABCD release v4.0, which became available several months after the fast‐track data. Tabulated study data from ABCD is also derived from ‘fast‐track’ fMRI images, using the ABCD Multi‐modal Processing Pipeline (MMPS; Hagler et al. 2019). The first ABCD condition in our analyses (‘Tabulated’) reflects participants whose MRI scans are represented in the tabulated data, which ABCD publications describe as excluding participants who did not have a complete T1 and/or rs‐fMRI scan which “passed visual inspection” (Hagler et al. 2019, 5; Figure  4). The second condition (“ABCD 4 Recommended”) includes participants who are recommended by DAIRC for rs‐fMRI analysis. DAIRC recommends exclusion from rs‐fMRI analysis if they fail segmentation and/or do not have 375 frames of resting state data with FD ≤ 0.2 mm (Hagler et al. 2019, 9; Figure  4).

Importantly, the ABCC dataset contained participants excluded from the ABCD pre‐tabulated and recommended subsets and vice versa. Subject counts for each condition, as well as non‐overlapping case counts, are shown in Figure 1.

### 1.4 Analytic Strategy

Two sets of logistic regression models predicting probability of exclusion within conditions were constructed. First, the unconditional effects of each demographic/behavioral predictor on missingness were considered by evaluating variables in separate models predicting the likelihood of a participant's resting state scan being excluded from analyses (136 models for each of 17 variables under 8 inclusion conditions). In a second set of conditional models, all predictors entered the model in a single step, again predicting likelihood of resting state scan exclusion (i.e., 8 models total). Significance values for neighborhood characteristics (ADI and COI) and general cognitive ability (matrix reasoning, NIH toolbox cognition composite, NIH toolbox crystallized intelligence composite) were Bonferroni corrected for multiple comparison.

Quality Control—Functional Connectivity plots (as in Ciric et al. 2017; Satterthwaite et al. 2012) were prepared as an index of data quality (i.e., presence of motion bias) within each of the ABCC conditions. First, connectivity matrices were calculated for all available subjects while scrubbing at each motion threshold (i.e., one per motion threshold, per participant). For each within‐subject pair‐wise correlation (connectivity) between regions‐of‐interest (ROIs), the correlation between each participant's mean FD and their connectivity in that pair was calculated as an index of the degree to which connectivity was related to motion. These “QC‐FC” values were then plotted against the Euclidean distance between the centers of the involved ROIs (in MNI space). Higher or lower QC‐FC for ROI‐pairs which are further apart (illustrated by a sloped rather than flat QC‐FC plot) provide evidence that functional connectivity in that pair is conflated with motion.

## 2 Results

For both the ABCC and ABCD conditions, a significant number of participants were excluded during pre‐processing steps conducted prior to motion correction. In ABCC data, 2,276 participants (19.2% of the total sample) were excluded prior to motion correction (see Figure 1). These excluded participants either had insufficient data for analysis after removing images marked as unusable in the operator QC report (“fastqc01”) or failed preprocessing by the ABCC pre‐processing pipeline. An additional 1093 participants (9.2%) were excluded between 0.5 mm and 0.2 mm of thresholding, and a final 3,257 participants (27%) were excluded with thresholding at 0.1 mm. In the ABCD v4.0 data, 521 participants (4.3% of ABCD) were excluded from the pre‐tabulated data. A further 1,728 participants (14.6%) were excluded based on DAIRC recommendations for inclusion in rs‐fMRI analysis. Importantly, the two processing streams were non‐overlapping: 1,426 participants not present in the DAIRC recommended condition were included in ABCC (before motion correction), and 1,802 participants included in the pre‐tabulated data were excluded from ABCC (see Figure 1).

Although missingness was most common in imaging data, substantial loss was present in both behavioral and demographic variables as well. More than 250 cases were missing values for child opportunity index (*n* = 1,093; 9.2%), household income (*n* = 1,018; 8.6%), area disadvantage index (*n* = 863; 7.27%), and NIH toolbox scores (*n* = 397; 3.34%).

### 2.1 Relation of Participant Motion to Demographic and Behavioral Variables

In order to characterize the relation of demographic and behavioral variables to participants' underlying motion, we present a correlation table of the continuous variables with FD within each motion scrubbing condition in Table 2, and a set of bivariate linear models between FD in each motion scrubbing condition and categorical variables in Table 3. Most variables showed a statistically significant relation with FD. Effects varied between trivial and moderate according to conventional criteria (*β* between 0.08 and 0.34 when *p* < 0.05). An analysis of the relation of participant characteristics to number of scrubbed volumes, by condition, is presented in the Supporting Information.

**TABLE 2:** Correlation of (continuous) study variables with rs‐fMRI motion (FD).

	FD at 0.1		FD at 0.2		FD at 0.3		FD at 0.4		FD at 0.5	
ADI	0.08	***	0.09	***	0.1	***	0.1	***	0.1	***
COI	−0.09	***	−0.11	***	−0.12	***	−0.12	***	−0.12	***
NIHTB flanker	−0.06	***	−0.06	***	−0.06	***	−0.06	***	−0.06	
NIHTB crystallized	−0.07	***	−0.08	***	−0.09	***	−0.09	***	−0.1	***
NIHTB total	−0.11	***	−0.13	***	−0.14	***	−0.14	***	−0.15	***
WISC V matrix	−0.08	***	−0.1	***	−0.11	***	−0.11	***	−0.11	***
P‐Factor	0.05	***	0.07	***	0.08	***	0.09	***	0.09	***
Internalizing	−0.04	***	−0.05	***	−0.05	***	−0.05	***	−0.05	***
Externalizing	0		0.01		0.01		0.01		0.01	
Age	−0.09	***	−0.1	***	−0.11	***	−0.11	***	−0.11	***
BMI	0.22	***	0.24	***	0.23	***	0.22	***	0.21	***

*** *p* < 0.001 in corresponding bivariate linear model.

**TABLE 3:** Relation of (categorical) study variables with rs‐fMRI motion (FD).

		0.1 mm		0.2 mm		0.3 mm		0.4 mm		0.5 mm	
Predictor	Contrast	*β*	*p*	*β*	*p*	*β*	*p*	*β*	*p*	*β*	*p*
Sex	M—F	0.24	< 0.001	0.23	< 0.001	0.22	< 0.001	0.19	< 0.001	0.13	< 0.001
Household income (ref: $100–200 k)	$0 to $25 k	0.34	< 0.001	0.34	< 0.001	0.34	< 0.001	0.32	< 0.001	0.28	< 0.001
	$25 to $50 k	0.15	< 0.001	0.15	< 0.001	0.14	< 0.001	0.14	< 0.001	0.11	0.01
	$50 to $75 k	0.16	< 0.001	0.16	< 0.001	0.16	< 0.001	0.18	< 0.001	0.16	< 0.001
	$75 to $100 k	0.01	1	0	1	0	1	0.01	0.997	0.01	0.98
	Over $200 k	−0.07	0.17	−0.07	0.16	−0.08	0.146	−0.07	0.173	−0.06	0.29
Parent education (ref: college education)	< High school	0.28	< 0.001	0.27	< 0.001	0.27	< 0.001	0.27	< 0.001	0.29	< 0.001
	High school	0.3	< 0.001	0.3	< 0.001	0.3	< 0.001	0.29	< 0.001	0.23	< 0.001
	Some college	0.17	< 0.001	0.17	< 0.001	0.17	< 0.001	0.16	< 0.001	0.13	< 0.001
	Graduate degree	−0.03	0.57	−0.03	0.71	−0.02	0.826	−0.01	0.957	0.01	0.93
Race/ethnicity (ref: white)	Black	0.3	< 0.001	0.31	< 0.001	0.33	< 0.001	0.34	< 0.001	0.32	< 0.001
	Hispanic	0.14	< 0.001	0.13	< 0.001	0.12	< 0.001	0.11	< 0.001	0.08	0.01
	Asian	0.02	0.99	0.01	1	−0.02	0.988	−0.03	0.951	−0.07	0.76
	Other	0.05	0.39	0.05	0.4	0.04	0.528	0.04	0.647	0.02	0.91
KSADs (ref: no exposures)	0–1	0.02	0.65	0.02	0.51	0.03	0.416	0.03	0.465	0.03	0.29
	> 2–0	0.13	< 0.001	0.13	< 0.001	0.13	< 0.001	0.13	< 0.001	0.11	0.01

*Note:* Contrasts from bivariate linear regression models. All corresponding *F* tests were significant at *p* < 0.01. Pubertal status is omitted as its *F* test was not significant in any condition (*p* > 0.05; see Supporting Information).

### 2.2 Relation of Exclusion to Demographic and Behavioral Variables

#### 2.2.1 Bivariate Models

**FIGURE 2:**
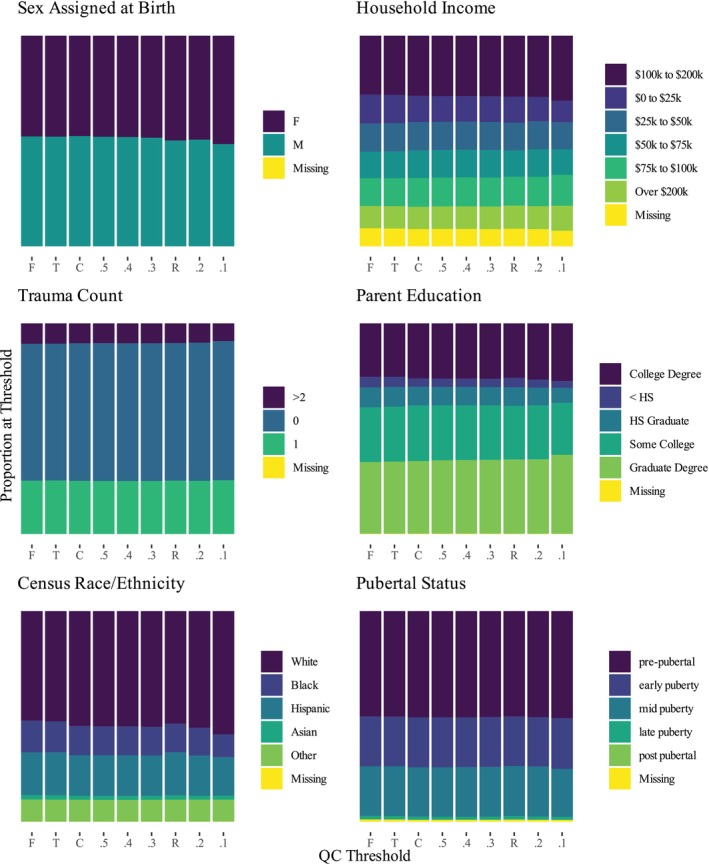
Categorical variables by inclusion criteria.

**FIGURE 3:**
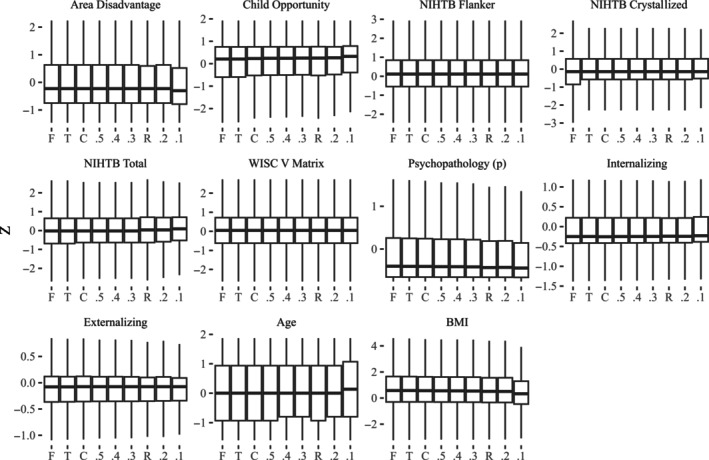
Continuous variables (standardized) by condition.

The distribution of demographic and behavioral variables across conditions is shown in Figures 2 and 3. In bivariate models, male sex, and internalizing psychopathology were associated with odds of missingness only in conditions involving some amount of motion correction (scrubbing at ≤ 0.3 mm; see Table 4). Externalizing psychopathology showed no evidence of a statistically significant association with odds of missingness. All other variables showed a statistically significant association with exclusion prior to motion filtering (i.e., in the tabulated and/or ABCC conditions). Exclusion patterns were particularly concerning in cases with multiple exclusion risk factors. For example, of the 552 participants in ABCD who identified as non‐white and who had a p‐factor score of greater than 1.5, 153 (28%) were flagged for exclusion in resting state analyses by the DAIRC (vs. 18.5% in the rest of the sample); of the 382 ABCD participants assigned male at birth with NIH toolbox scores of z ≤ − 1.5, 150 (39%) were not recommended for inclusion by DAIRC (vs. 18.3% in the rest of the sample).

**TABLE 4:** Odds of exclusion—bivariate logistic regression models.

Condition	*T*	*C*	0.5	0.4	0.3	*R*	0.2	0.1
Variable	OR	OR	OR	OR	OR	OR	OR	OR
	[90% CI]	[90% CI]	[90% CI]	[90% CI]	[90% CI]	[90% CI]	[90% CI]	[90% CI]
	*p*	*p*	*p*	*p*	*p*	*p*	*p*	*p*
Sex (male)	1.11	0.96	1.04	1.06	1.11*	1.52***	1.24***	1.30***
	[0.93–1.33]	[0.88–1.06]	[0.95–1.13]	[0.97–1.15]	[1.02–1.21]	[1.38–1.67]	[1.15–1.35]	[1.21–1.40]
	0.237	0.443	0.435	0.211	0.014	< 0.001	< 0.001	< 0.001
Household income (ref: $100–$200 k)								
$0–$25 k	1.87***	1.68***	1.82***	1.84***	1.82***	1.98***	1.92***	1.95***
	[1.43–2.44]	[1.46–1.94]	[1.58–2.08]	[1.61–2.10]	[1.59–2.07]	[1.72–2.29]	[1.69–2.17]	[1.73–2.21]
	< 0.001	< 0.001	< 0.001	< 0.001	< 0.001	< 0.001	< 0.001	< 0.001
$25–$50 k	1.10	1.09	1.13	1.15	1.18*	1.28**	1.17*	1.29***
	[0.80–1.49]	[0.93–1.27]	[0.97–1.31]	[0.99–1.33]	[1.02–1.36]	[1.10–1.50]	[1.02–1.34]	[1.15–1.46]
	0.550	0.292	0.112	0.069	0.025	0.002	0.023	< 0.001
$50–$75 k	1.05	0.92	0.97	0.98	0.99	1.29**	1.04	1.32***
	[0.76–1.44]	[0.78–1.09]	[0.83–1.14]	[0.84–1.14]	[0.85–1.15]	[1.10–1.51]	[0.90–1.19]	[1.17–1.50]
	0.750	0.336	0.747	0.795	0.893	0.002	0.581	< 0.001
$75–$100 k	1.11	0.92	0.95	0.94	0.98	1.03	0.95	0.99
	[0.81–1.51]	[0.78–1.08]	[0.81–1.10]	[0.81–1.10]	[0.84–1.13]	[0.87–1.21]	[0.82–1.09]	[0.88–1.12]
	0.506	0.304	0.490	0.455	0.767	0.732	0.434	0.881
> $200 k	0.95	1.10	1.07	1.06	1.06	0.97	1.01	0.96
	[0.67–1.34]	[0.93–1.30]	[0.91–1.26]	[0.90–1.24]	[0.90–1.24]	[0.81–1.16]	[0.87–1.17]	[0.84–1.09]
	0.795	0.268	0.422	0.495	0.502	0.715	0.927	0.494
Highest parental education (ref: college degree)								
< High school	1.59*	2.59***	2.56***	2.59***	2.51***	1.65***	2.52***	2.21***
	[1.10–2.25]	[2.13–3.14]	[2.12–3.10]	[2.14–3.12]	[2.08–3.02]	[1.34–2.02]	[2.10–3.02]	[1.83–2.68]
	0.011	< 0.001	< 0.001	< 0.001	< 0.001	< 0.001	< 0.001	< 0.001
HS grad.	1.25	1.59***	1.65***	1.65***	1.69***	1.59***	1.73***	1.86***
	[0.92–1.69]	[1.34–1.87]	[1.41–1.94]	[1.41–1.94]	[1.45–1.98]	[1.35–1.87]	[1.49–2.00]	[1.61–2.14]
	0.152	< 0.001	< 0.001	< 0.001	< 0.001	< 0.001	< 0.001	< 0.001
Some college	0.86	1.07	1.13	1.15*	1.17*	1.20**	1.25***	1.26***
	[0.67–1.10]	[0.94–1.22]	[1.00–1.28]	[1.02–1.30]	[1.04–1.32]	[1.06–1.37]	[1.12–1.40]	[1.14–1.40]
	0.236	0.330	0.054	0.027	0.011	0.005	< 0.001	< 0.001
Graduate	0.83	1.08	1.04	1.03	1.03	0.90	1.05	0.96
	[0.65–1.04]	[0.95–1.22]	[0.92–1.17]	[0.92–1.16]	[0.92–1.16]	[0.79–1.02]	[0.94–1.17]	[0.87–1.05]
	0.109	0.227	0.545	0.620	0.597	0.085	0.417	0.380
Census race/ethnicity (ref: White)								
Black	1.69***	1.85***	1.87***	1.86***	1.85***	1.79***	1.89***	2.11***
	[1.34–2.13]	[1.63–2.10]	[1.66–2.12]	[1.65–2.10]	[1.64–2.09]	[1.58–2.02]	[1.69–2.12]	[1.89–2.36]
	< 0.001	< 0.001	< 0.001	< 0.001	< 0.001	< 0.001	< 0.001	< 0.001
Hispanic	1.08	1.71***	1.61***	1.60***	1.61***	1.13	1.54***	1.49***
	[0.85–1.37]	[1.52–1.92]	[1.44–1.81]	[1.43–1.79]	[1.44–1.80]	[1.00–1.27]	[1.38–1.70]	[1.36–1.65]
	0.513	< 0.001	< 0.001	< 0.001	< 0.001	0.057	< 0.001	< 0.001
Asian	1.72*	2.22***	2.09***	2.02***	2.12***	1.42*	2.01***	1.48**
	[0.98–2.80]	[1.67–2.94]	[1.58–2.74]	[1.53–2.65]	[1.61–2.76]	[1.04–1.91]	[1.54–2.60]	[1.15–1.92]
	0.043	< 0.001	< 0.001	< 0.001	< 0.001	0.023	< 0.001	0.003
Other	1.26	1.39***	1.41***	1.42***	1.37***	1.20*	1.30***	1.27***
	[0.93–1.67]	[1.19–1.63]	[1.22–1.63]	[1.22–1.64]	[1.19–1.58]	[1.03–1.40]	[1.13–1.48]	[1.13–1.44]
	0.126	< 0.001	< 0.001	< 0.001	< 0.001	0.019	< 0.001	< 0.001
KSADS trauma count (ref: 0 exposures)								
1 Trauma	0.92	1.04	1.06	1.05	1.06	1.03	1.04	1.02
	[0.74–1.13]	[0.94–1.16]	[0.95–1.17]	[0.95–1.17]	[0.96–1.17]	[0.92–1.14]	[0.95–1.15]	[0.93–1.11]
	0.435	0.458	0.281	0.316	0.239	0.646	0.374	0.697
≥ 2 Trauma	1.18	1.18*	1.19*	1.15	1.15*	1.25**	1.27***	1.32***
	[0.88–1.55]	[1.01–1.37]	[1.03–1.38]	[0.99–1.33]	[1.00–1.33]	[1.07–1.45]	[1.11–1.45]	[1.16–1.50]
	0.264	0.032	0.019	0.058	0.049	0.004	0.001	< 0.001
Pubertal status (ref: pre‐pubertal)								
Early puberty	1.16	1.05	1.05	1.04	1.02	1.04	1.06	1.01
	[0.93–1.43]	[0.94–1.18]	[0.94–1.17]	[0.93–1.16]	[0.91–1.13]	[0.93–1.16]	[0.96–1.17]	[0.92–1.10]
	0.186	0.384	0.352	0.465	0.744	0.526	0.257	0.883
Mid puberty	1.00	1.07	1.06	1.04	1.01	0.96	0.99	1.10*
	[0.80–1.25]	[0.96–1.20]	[0.95–1.18]	[0.94–1.16]	[0.91–1.13]	[0.85–1.07]	[0.89–1.09]	[1.00–1.20]
	0.991	0.231	0.285	0.449	0.831	0.453	0.825	0.044
Late puberty	1.02	1.74***	1.84***	1.81***	1.65**	1.02	1.54**	1.23
	[0.46–1.96]	[1.25–2.39]	[1.34–2.50]	[1.32–2.46]	[1.20–2.24]	[0.69–1.46]	[1.13–2.07]	[0.92–1.66]
	0.958	0.001	< 0.001	< 0.001	0.002	0.921	0.005	0.167
Post pubertal	4.59*	0.89	1.27	1.23	1.12	3.07	1.85	2.45
	[0.70–17.52]	[0.14–3.37]	[0.28–4.28]	[0.27–4.13]	[0.25–3.76]	[0.91–9.64]	[0.55–5.80]	[0.73–11.07]
	0.050	0.877	0.716	0.757	0.866	0.056	0.294	0.178
ADI	1.05	0.95	0.99	1.00	1.01	1.16***	1.04	1.13***
	[0.96–1.15]	[0.91–1.00]	[0.94–1.04]	[0.95–1.04]	[0.97–1.06]	[1.11–1.21]	[1.00–1.08]	[1.09–1.18]
	0.632	0.076	1.288	1.877	1.246	< 0.001	0.113	< 0.001
COI	0.91	0.85***	0.83***	0.82***	0.82***	0.81***	0.82***	0.79***
	[0.83–0.99]	[0.81–0.89]	[0.79–0.87]	[0.79–0.86]	[0.78–0.85]	[0.77–0.85]	[0.78–0.85]	[0.76–0.82]
	0.061	< 0.001	< 0.001	< 0.001	< 0.001	< 0.001	< 0.001	< 0.001
NIHTB flanker	0.77***	0.95*	0.93**	0.93***	0.92***	0.85***	0.91***	0.89***
	[0.70–0.85]	[0.91–1.00]	[0.89–0.97]	[0.89–0.97]	[0.88–0.96]	[0.81–0.89]	[0.88–0.95]	[0.86–0.92]
	< 0.001	0.042	0.002	0.001	< 0.001	< 0.001	< 0.001	< 0.001
NIHTB crystallized	0.76***	0.89***	0.86***	0.85***	0.85***	0.77***	0.83***	0.83***
	[0.69–0.84]	[0.84–0.93]	[0.82–0.90]	[0.82–0.89]	[0.81–0.89]	[0.74–0.81]	[0.80–0.87]	[0.80–0.86]
	< 0.001	< 0.001	< 0.001	< 0.001	< 0.001	< 0.001	< 0.001	< 0.001
NIHTB total	0.71***	0.88***	0.84***	0.83***	0.82***	0.72***	0.80***	0.78***
	[0.65–0.78]	[0.84–0.92]	[0.80–0.88]	[0.79–0.87]	[0.78–0.86]	[0.68–0.75]	[0.77–0.83]	[0.75–0.81]
	< 0.001	< 0.001	< 0.001	< 0.001	< 0.001	< 0.001	< 0.001	< 0.001
WISC V matrix	0.76***	0.92***	0.90***	0.89***	0.87***	0.77***	0.85***	0.83***
	[0.69–0.83]	[0.88–0.96]	[0.86–0.94]	[0.85–0.93]	[0.84–0.91]	[0.74–0.81]	[0.82–0.88]	[0.80–0.86]
	< 0.001	0.001	< 0.001	< 0.001	< 0.001	< 0.001	< 0.001	< 0.001
P‐factor	1.14**	1.04	1.08***	1.09***	1.10***	1.20***	1.13***	1.13***
	[1.05–1.23]	[1.00–1.09]	[1.04–1.13]	[1.04–1.13]	[1.06–1.15]	[1.16–1.26]	[1.09–1.17]	[1.09–1.17]
	0.001	0.070	< 0.001	< 0.001	< 0.001	< 0.001	< 0.001	< 0.001
Internalizing	0.97	0.99	0.97	0.96	0.95*	0.92***	0.95*	0.91***
	[0.88–1.06]	[0.94–1.03]	[0.92–1.01]	[0.92–1.00]	[0.91–0.99]	[0.88–0.97]	[0.91–0.99]	[0.88–0.95]
	0.469	0.570	0.124	0.067	0.016	0.001	0.012	< 0.001
Externalizing	1.03	0.98	0.99	0.99	1.00	1.01	1.00	1.02
	[0.94–1.12]	[0.94–1.03]	[0.95–1.04]	[0.95–1.03]	[0.96–1.05]	[0.96–1.06]	[0.96–1.04]	[0.98–1.06]
	0.575	0.496	0.810	0.624	0.860	0.670	0.843	0.313
Age	0.89**	0.88***	0.86***	0.85***	0.84***	0.82***	0.83***	0.82***
	[0.81–0.97]	[0.84–0.92]	[0.82–0.90]	[0.82–0.89]	[0.81–0.88]	[0.79–0.86]	[0.80–0.87]	[0.79–0.86]
	0.009	< 0.001	< 0.001	< 0.001	< 0.001	< 0.001	< 0.001	< 0.001
BMI	1.04	1.05**	1.07***	1.07***	1.09***	1.22***	1.14***	1.25***
	[0.98–1.10]	[1.02–1.08]	[1.04–1.10]	[1.04–1.11]	[1.06–1.12]	[1.18–1.26]	[1.11–1.18]	[1.22–1.28]
	0.200	0.002	< 0.001	< 0.001	< 0.001	< 0.001	< 0.001	< 0.001

#### 2.2.2 Adjusted Models

Odds ratios from adjusted models are listed in Table 5. Across ABCC conditions, exclusion was more likely for participants identified as Black, who had lower scores on the Area Deprivation Index, or who had lower scores on the Child Opportunity Index. In all conditions, or all conditions but pre‐tabulated data, exclusion was more likely for participants with higher general psychopathology, lower externalizing psychopathology factor, Asian Race/Ethnicity, and younger age. In a third set of variables, Exclusion was more likely once motion thresholding above a certain stringency was applied. These included male sex (scrubbing at 0.3 mm or below), higher BMI (0.3 mm), lower NIH toolbox total score (0.5 mm), higher NIH toolbox crystallized score (0.1 mm), and trauma count ≥ 2 (0.1 mm). Exclusion was more likely in ABCC conditions for participants with race/ethnicity identifications of Hispanic or Other, late puberty status, and parent education (< HS), but these associations became non‐significant as the motion scrubbing threshold was lowered. Lower flanker task performance, lower WISC V Matrix performance, lower internalizing psychopathology, and parent education (all levels but < High School) were related to odds of exclusion in some but not all conditions without a clear trend. In adjusted models, only household income showed no significant associations with exclusion in any condition.

**TABLE 5:** Odds of exclusion—adjusted (within condition) logistic regression models.

Condition	*T*	*C*	0.5	0.4	0.3	*R*	0.2	0.1
Variable	OR	OR	OR	OR	OR	OR	OR	OR
	[90% CI]	[90% CI]	[90% CI]	[90% CI]	[90% CI]	[90% CI]	[90% CI]	[90% CI]
	*p*	*p*	*p*	*p*	*p*	*p*	*p*	*p*
Intercept	0.04***	0.16***	0.18***	0.19***	0.20***	0.14***	0.25***	0.81**
	[0.03–0.05]	[0.13–0.19]	[0.15–0.22]	[0.16–0.22]	[0.17–0.24]	[0.12–0.17]	[0.21–0.29]	[0.70–0.93]
	0.000	0.000	0.000	0.000	0.000	0.000	0.000	0.003
Sex (male)	0.99	0.99	1.07	1.10	1.15*	1.48***	1.26***	1.32***
	[0.78–1.27]	[0.88–1.13]	[0.95–1.21]	[0.97–1.24]	[1.02–1.30]	[1.30–1.69]	[1.13–1.41]	[1.19–1.46]
	0.945	0.935	0.264	0.127	0.019	0.000	0.000	0.000
Household income (ref: $100–$200 k)								
$0–$25 k	1.41	1.09	1.14	1.16	1.10	1.17	1.11	1.03
	[0.91–2.17]	[0.86–1.37]	[0.92–1.42]	[0.93–1.44]	[0.89–1.37]	[0.93–1.47]	[0.91–1.36]	[0.85–1.25]
	0.125	0.477	0.229	0.181	0.368	0.180	0.293	0.746
$25–$50 k	0.87	0.91	0.89	0.91	0.91	0.90	0.86	0.89
	[0.57–1.32]	[0.73–1.12]	[0.73–1.09]	[0.74–1.11]	[0.75–1.11]	[0.73–1.11]	[0.72–1.03]	[0.75–1.04]
	0.525	0.357	0.252	0.345	0.367	0.341	0.112	0.145
$50–$75 k	1.04	0.91	0.90	0.91	0.91	1.06	0.92	1.08
	[0.71–1.50]	[0.74–1.10]	[0.75–1.09]	[0.75–1.09]	[0.76–1.09]	[0.88–1.28]	[0.78–1.09]	[0.93–1.25]
	0.836	0.313	0.288	0.289	0.295	0.552	0.323	0.296
$75–$100 k	1.06	0.94	0.96	0.95	0.98	0.99	0.93	0.92
	[0.74–1.49]	[0.78–1.12]	[0.80–1.13]	[0.80–1.13]	[0.83–1.16]	[0.82–1.18]	[0.80–1.09]	[0.80–1.05]
	0.746	0.474	0.602	0.593	0.810	0.875	0.378	0.201
> $200 k	1.04	0.95	0.98	0.98	1.00	1.10	0.98	0.96
	[0.70–1.52]	[0.79–1.15]	[0.82–1.18]	[0.82–1.18]	[0.83–1.19]	[0.90–1.34]	[0.82–1.16]	[0.83–1.12]
	0.834	0.619	0.842	0.864	0.968	0.358	0.798	0.613
Highest parental education (ref: college degree)								
< High school	0.97	1.43*	1.48*	1.49**	1.43*	1.07	1.46**	1.30
	[0.53–1.71]	[1.05–1.95]	[1.10–1.99]	[1.11–2.00]	[1.06–1.91]	[0.77–1.47]	[1.10–1.93]	[0.98–1.73]
	0.910	0.024	0.010	0.008	0.017	0.681	0.009	0.070
HS grad.	1.09	1.19	1.25	1.24	1.25	1.18	1.25*	1.16
	[0.70–1.67]	[0.94–1.52]	[0.99–1.57]	[0.98–1.55]	[1.00–1.56]	[0.93–1.49]	[1.01–1.55]	[0.95–1.42]
	0.713	0.152	0.062	0.068	0.051	0.173	0.037	0.145
Some college	0.69*	0.98	1.02	1.03	1.03	1.00	1.09	1.01
	[0.49–0.97]	[0.82–1.16]	[0.86–1.20]	[0.87–1.21]	[0.88–1.20]	[0.84–1.18]	[0.94–1.26]	[0.89–1.15]
	0.033	0.781	0.825	0.765	0.755	0.984	0.255	0.863
Graduate	1.06	1.16*	1.13	1.12	1.13	1.02	1.14*	1.05
	[0.80–1.41]	[1.01–1.35]	[0.98–1.30]	[0.98–1.29]	[0.98–1.29]	[0.88–1.19]	[1.00–1.30]	[0.94–1.17]
	0.676	0.043	0.091	0.104	0.090	0.780	0.047	0.406
Census race/ethnicity (ref: White)								
Black	1.36	1.85***	1.68***	1.62***	1.57***	1.20	1.51***	1.46***
	[0.94–1.97]	[1.52–2.26]	[1.39–2.02]	[1.34–1.96]	[1.31–1.89]	[0.98–1.45]	[1.27–1.79]	[1.23–1.72]
	0.100	0.000	0.000	0.000	0.000	0.070	0.000	0.000
Hispanic	1.04	1.32***	1.19*	1.18*	1.19*	0.87	1.11	1.09
	[0.75–1.44]	[1.12–1.55]	[1.01–1.39]	[1.01–1.38]	[1.02–1.38]	[0.74–1.03]	[0.96–1.28]	[0.96–1.24]
	0.798	0.001	0.032	0.036	0.025	0.116	0.160	0.191
Asian	2.10*	1.89***	1.93***	1.88***	2.05***	1.87***	1.98***	1.52**
	[1.11–3.67]	[1.34–2.62]	[1.38–2.65]	[1.35–2.59]	[1.49–2.81]	[1.30–2.64]	[1.45–2.68]	[1.13–2.06]
	0.015	0.000	0.000	0.000	0.000	0.001	0.000	0.007
Other	1.20	1.37***	1.34**	1.34***	1.28**	0.99	1.17	1.11
	[0.83–1.69]	[1.14–1.64]	[1.12–1.59]	[1.13–1.59]	[1.08–1.51]	[0.81–1.19]	[0.99–1.37]	[0.96–1.28]
	0.319	0.001	0.001	0.001	0.005	0.887	0.060	0.167
KSADS trauma count (ref: 0 exposures)								
1 Trauma	0.92	1.03	1.05	1.05	1.05	1.02	1.03	0.96
	[0.71–1.19]	[0.91–1.17]	[0.93–1.19]	[0.93–1.18]	[0.93–1.18]	[0.89–1.15]	[0.92–1.15]	[0.87–1.06]
	0.537	0.645	0.417	0.463	0.432	0.814	0.663	0.464
≥ 2 Trauma	1.18	1.18	1.12	1.08	1.06	1.02	1.13	1.20*
	[0.83–1.66]	[0.98–1.42]	[0.94–1.34]	[0.90–1.29]	[0.89–1.27]	[0.84–1.22]	[0.96–1.33]	[1.03–1.40]
	0.348	0.086	0.210	0.391	0.504	0.875	0.141	0.021
Pubertal status (ref: pre‐pubertal)								
Early puberty	1.14	1.01	1.02	1.02	0.99	1.03	1.04	1.00
	[0.87–1.48]	[0.88–1.16]	[0.89–1.16]	[0.89–1.16]	[0.87–1.13]	[0.89–1.18]	[0.92–1.17]	[0.89–1.11]
	0.330	0.881	0.822	0.774	0.938	0.691	0.568	0.975
Mid puberty	0.97	1.01	1.03	1.03	1.03	1.04	1.01	1.08
	[0.70–1.34]	[0.86–1.20]	[0.88–1.21]	[0.88–1.21]	[0.88–1.21]	[0.88–1.24]	[0.87–1.17]	[0.95–1.24]
	0.866	0.872	0.714	0.684	0.675	0.639	0.876	0.241
Late puberty	1.13	1.60*	1.67*	1.65*	1.53*	1.00	1.45	1.03
	[0.46–2.41]	[1.05–2.40]	[1.11–2.47]	[1.10–2.45]	[1.02–2.26]	[0.62–1.57]	[0.98–2.12]	[0.71–1.49]
	0.770	0.024	0.011	0.013	0.034	1.000	0.056	0.892
Post pubertal	3.32	0.51	0.45	0.44	0.42	2.29	1.32	0.96
	[0.17–19.69]	[0.03–3.01]	[0.02–2.60]	[0.02–2.58]	[0.02–2.41]	[0.46–9.62]	[0.26–5.51]	[0.23–4.77]
	0.271	0.536	0.456	0.452	0.419	0.266	0.710	0.959
Area disadvantage	1.01	0.67***	0.70***	0.72***	0.73***	0.97	0.76***	0.85***
	[0.85–1.20]	[0.61–0.73]	[0.64–0.77]	[0.66–0.78]	[0.67–0.79]	[0.89–1.07]	[0.70–0.83]	[0.79–0.92]
	0.466	0.000	0.000	0.000	0.000	0.288	0.000	0.000
Child opportunity	1.12	0.77***	0.79***	0.80***	0.80***	0.97	0.83***	0.85***
	[0.92–1.37]	[0.70–0.85]	[0.72–0.86]	[0.73–0.88]	[0.73–0.87]	[0.88–1.08]	[0.76–0.90]	[0.79–0.93]
	0.123	0.000	0.000	0.000	0.000	0.311	0.000	0.000
NIHTB flanker	0.86*	1.01	1.02	1.01	1.02	0.99	1.02	1.01
	[0.74–0.99]	[0.94–1.08]	[0.95–1.09]	[0.95–1.09]	[0.96–1.10]	[0.92–1.06]	[0.96–1.09]	[0.95–1.07]
	0.036	0.804	0.607	0.698	0.489	0.737	0.502	0.750
NIHTB crystallized	0.92	1.01	1.05	1.05	1.08	1.09	1.08	1.14**
	[0.73–1.16]	[0.90–1.13]	[0.94–1.18]	[0.94–1.17]	[0.97–1.20]	[0.96–1.22]	[0.98–1.20]	[1.04–1.25]
	0.495	0.858	0.353	0.397	0.159	0.174	0.121	0.004
NIHTB total	0.92	0.95	0.88*	0.88*	0.85**	0.79***	0.84**	0.82***
	[0.70–1.19]	[0.83–1.08]	[0.77–1.00]	[0.78–1.01]	[0.75–0.96]	[0.69–0.90]	[0.75–0.95]	[0.74–0.91]
	0.263	0.214	0.029	0.031	0.005	0.000	0.002	0.000
WISC V matrix	0.84**	1.00	1.00	0.99	0.98	0.89***	0.96	0.96*
	[0.74–0.94]	[0.94–1.07]	[0.95–1.07]	[0.94–1.05]	[0.93–1.04]	[0.84–0.95]	[0.91–1.01]	[0.91–1.00]
	0.002	0.454	0.442	0.402	0.295	0.000	0.051	0.035
Psychopathology	1.10	1.08*	1.11***	1.11***	1.11***	1.16***	1.13***	1.11***
	[0.99–1.22]	[1.02–1.14]	[1.05–1.17]	[1.05–1.17]	[1.06–1.17]	[1.10–1.22]	[1.08–1.19]	[1.06–1.17]
	0.059	0.012	0.000	0.000	0.000	0.000	0.000	0.000
Internalizing	1.01	0.99	0.98	0.97	0.96	0.93*	0.95	0.92***
	[0.91–1.13]	[0.94–1.05]	[0.92–1.03]	[0.92–1.02]	[0.91–1.01]	[0.88–0.98]	[0.91–1.00]	[0.87–0.96]
	0.809	0.788	0.380	0.237	0.118	0.013	0.058	0.000
Externalizing	0.96	0.94*	0.94*	0.94*	0.94*	0.94*	0.94**	0.93**
	[0.87–1.07]	[0.89–1.00]	[0.89–0.99]	[0.89–0.99]	[0.90–0.99]	[0.89–0.99]	[0.89–0.98]	[0.89–0.98]
	0.479	0.040	0.027	0.019	0.028	0.019	0.009	0.004
Age	0.90	0.88***	0.85***	0.85***	0.84***	0.82***	0.82***	0.82***
	[0.81–1.00]	[0.83–0.93]	[0.81–0.90]	[0.80–0.89]	[0.79–0.88]	[0.77–0.86]	[0.78–0.86]	[0.79–0.86]
	0.060	0.000	0.000	0.000	0.000	0.000	0.000	0.000
BMI	1.00	1.01	1.03	1.02	1.04*	1.16***	1.09***	1.18***
	[0.93–1.07]	[0.97–1.05]	[0.99–1.07]	[0.99–1.06]	[1.00–1.08]	[1.12–1.21]	[1.05–1.12]	[1.14–1.22]
	0.901	0.748	0.170	0.199	0.047	0.000	0.000	0.000

##### 2.2.2.1 Sensitivity of Adjusted Models to Site Control

**FIGURE 4:**
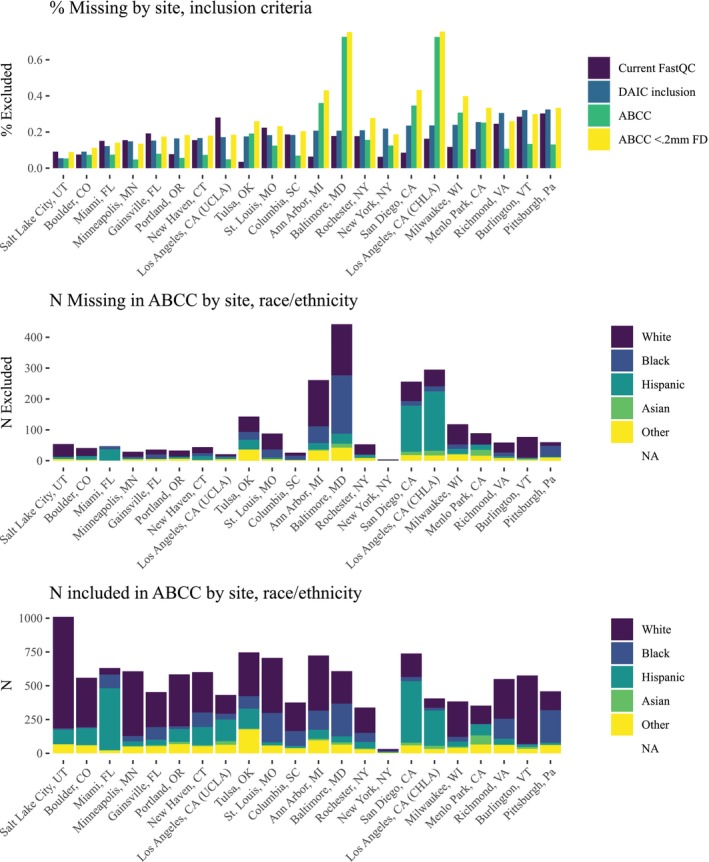
Some sites showed higher rates of exclusion in ABCC conditions versus ABCD Recommended, and some of those sites (e.g., LA, Baltimore, San Diego) had higher numbers of non‐white participants.

Scanner model was not significantly associated with odds of exclusion after site was controlled for (all *p* > 0.978; see Table S1). In models including collection site (but not scanner), we no longer found evidence of an association with census race/ethnicity (except for Asian), ADI, or COI with odds of exclusion (see Supporting Information). In subsequent exploratory analyses, we found that the ABCC conditions showed more variability in missingness with site than the pre‐tabulated or DAIRC conditions (up to 76% of participants missing in some sites, vs. a max of 32% in DAIRC) prior to motion correction, and that several sites with higher rates of exclusion in ABCC had greater percentages of racial/ethnic minority participants (see Figure 4).

#### 2.2.3 Trends in Effect Size Across Scrubbing Thresholds

**FIGURE 5:**
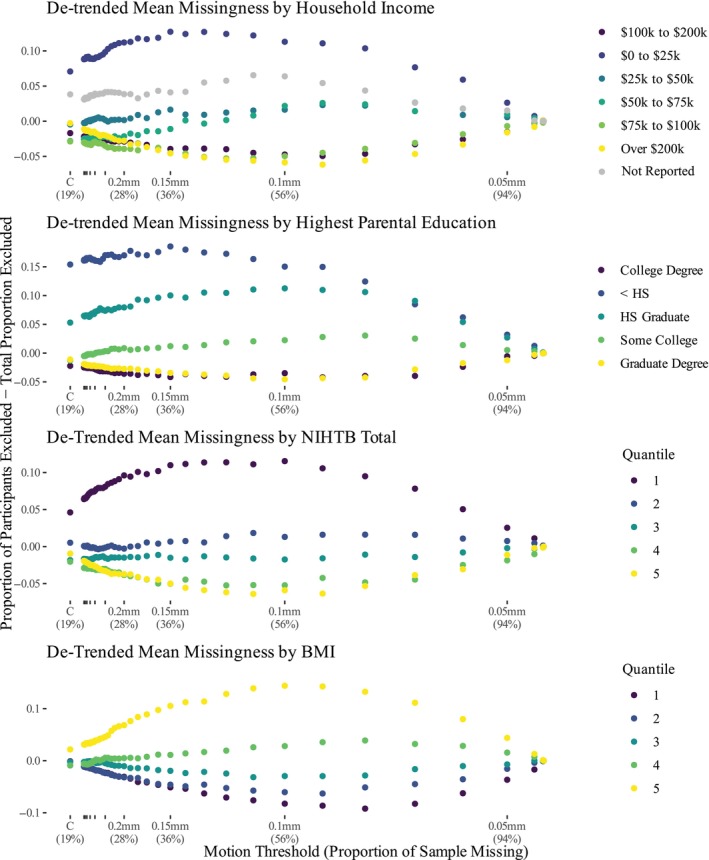
The proportion of cases missing in excess of the sample average (y axis) against the percentage of data missing from the sample (with conditions labeled—x axis). The steepest gains in bias occur early in thresholding—as more data is excluded, biases decelerate and then self‐correct below around 0.1 mm thresholding. Additional variables are plotted in the Supporting Information.

We originally hypothesized that, during scrubbing, differences between included and excluded participants would become larger as the motion threshold was decreased. Visual inspection of the data did not support this hypothesis. Instead, differences between included and excluded participants emerged prior to motion thresholding and increased in a roughly linear pattern until the scrubbing threshold reached approximately 0.2 mm (28% of data excluded), whereupon differences began to decrease (see Figure 5 for a representative subset of these trends). This pattern follows naturally given that almost all participants were excluded by scrubbing at a sufficiently strict threshold—participants who were more likely to be excluded were disproportionately excluded at more liberal thresholds, leaving fewer such participants to be excluded at more stringent thresholds (at the extreme, once all or nearly all participants are excluded, exclusion was equally likely for all participants). Additional figures, together with the originally planned omnibus model, are presented in the Supporting Information.

### 2.3 Motion Artifact

**FIGURE 6:**
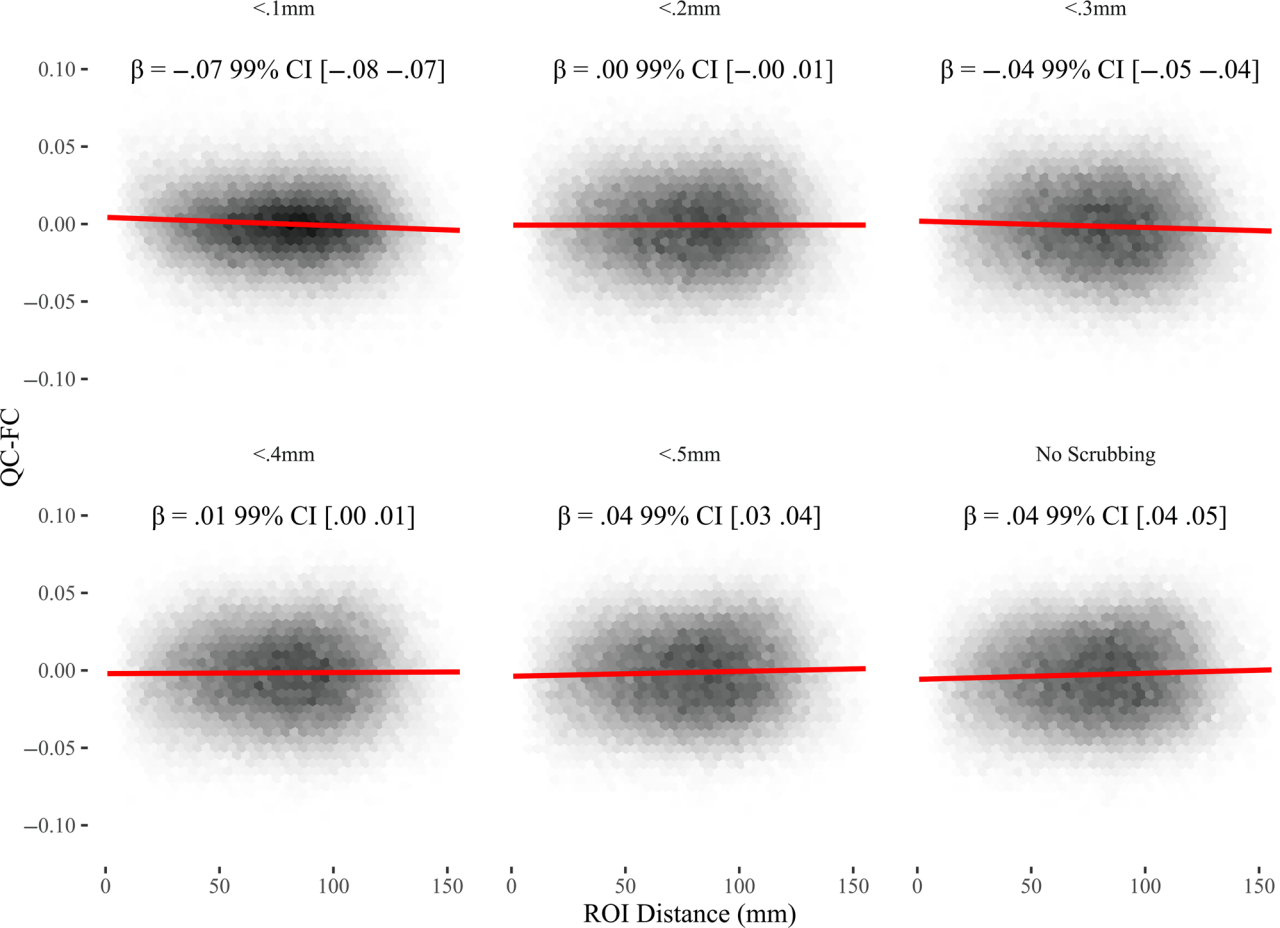
Hexagonal binned density plots of the correlation between participants' functional connectivity in each pair of regions in the HCP 2016 cortical atlas and FD (QC‐FC), plotted against the average Euclidean distance between said regions. Data from all ABCC participants is included (*n* = 9600).

QC‐FC plots are presented in Figure 6. Motion Scrubbing at 0.2 mm (*β* = 0, 99% CI [0–0.01]) and 0.4 mm (*β* = 0.01, 99% CI [0–0.01]) showed the least evidence of motion‐artifact by this metric. Motion scrubbing at 0.5 mm (or no scrubbing) revealed a pattern where higher motion participants had stronger correlations in distant ROI pairs (evidence of motion bias). Motion scrubbing at 0.1 mm or 0.3 mm showed evidence of increased distance dependence in the opposite direction.

## 3 Discussion

Investigators working with fMRI data must balance validity threats from inclusion of poor‐quality data against those created by listwise deletion—there is little available guidance on how best to do this. The resulting heterogeneity in QC procedures is a threat to the reproducibility and clarity of the fMRI literature. We set out to explore the impact of a variety of QC conditions on the composition of the ABCD sample in order to provide concrete guidance to researchers. We found that the cleaned ABCD sample varied in size and character depending on which QC pathway was selected. Across conditions, as few as 4.3% and as many as 44.2% of the sample was excluded. Excluded participants differed from included participants across a range of characteristics in both bivariate and adjusted models. Differences between excluded and included participants were evident before motion scrubbing and/or following motion scrubbing at thresholds more liberal than recommended by either the DAIRC and/or our own quality control figures (i.e., ≥ 0.3 mm). Participants were more likely to be excluded during QC who had lower household income, lower parent education, non‐white race/ethnicity, trauma exposure, male sex, and lower neighborhood opportunity as well as lower scores on cognitive performance measures, higher general psychopathology, younger age, and higher BMI. That is, exclusion was generally more likely for participants who were from more marginalized social groups, at higher risk of poor behavioral and cognitive outcomes, and who scored lower on measures of those outcomes. These results indicate that, at least in this sample, there is no “sweet spot” of QC stringency at which effects of motion artifacts are minimized and excluded data are missing completely at random. Nevertheless, a number of recommendations follow from our results.

### 3.1 Recommendations for Data Analysis

Our results indicate that QC‐related exclusions from rs‐fMRI datasets are not missing completely at random. Statistical techniques are available which can minimize biases introduced by this pattern of missingness. Best practices for handling this missing data are dependent on investigators' research question and what variables they intend to use.

#### 3.1.1 When Study Variables Are Unrelated to Exclusion

If an investigator is interested in the relation of resting‐state brain activity and some number of other variables unrelated to odds of exclusion, QC‐related missingness will still impact the generalizability of findings as the remaining sample will no longer be as representative of the U.S. population as the original sample (i.e., there will be a selection bias). This pattern can contribute to the under‐representation of marginalized and/or minority populations in neuroscience and yield findings which principally describe majority groups (Ricard et al. 2023). Selection bias can plausibly be corrected by population weighting (Gard et al. 2023). However, in our investigation very few variables were unrelated to missingness under adequate quality control, and investigators should explicitly test the relation of their study variables to missingness before relying on population weighting alone.

#### 3.1.2 When Study Variables Are Related to Exclusion

In the most common case, when investigators are interested in the relation of rs‐fMRI signal to other variables which are associated with rs‐fMRI missingness, some form of multivariate missing data handling procedure (e.g., multiple imputation [MI] or full‐information maximum likelihood modeling [FIML]) is recommended (Harrell 2015; Woods et al. 2023). This is due to the volume of missing data (19% after even liberal motion scrubbing) and its relation to other variables. Both approaches are well established and have relative strengths and weaknesses (Lee and Shi 2021; Woods et al. 2023). Correctly employed, they should minimize the impact of missing data on study results.

#### 3.1.3 Limitations of Missing Data Handling

An important additional concern is that missingness of rs‐fMRI data may be driven by characteristics of the resting brain, itself., that is, there may be one or more brain connectivity phenotypes which are more likely to produce participant motion in the scanner, resulting in participant exclusion. Indeed, a study of 118 adults found that motion was related to brain connectivity differences between high and low motion participants but not between high and low motion scans collected on the same participant—suggesting that the motion related signal might reflect a stable neural phenotype of participants who are prone to motion (Zeng et al. 2014). If exclusion related to QC is causally related to brain connectivity differences, then missing rsfMRI data will be formally missing not at random (MNAR). While procedures such as MI may reduce the size of biases resulting from analysis of MNAR data (Woods et al. 2023), such biases are not fully correctable by mathematical correction. Because any brain signal that contributes to participant exclusion is necessarily unobservable, it is not possible to directly test whether the data are MNAR. However, the broad association of participant characteristics (themselves related to brain signal) with missingness that we have described suggest that some data is MNAR. Further work may leverage the longitudinal design of studies such as the ABCD and/or examine participants just above exclusion thresholds to further explore the relation between exclusion and brain‐signal.

Importantly, these findings should not be taken to recommend the inclusion of poor‐quality data in analysis. Including poor quality data may create a different sort of bias, equally serious, wherein participant characteristics are associated with brain‐signal artifacts (Kay et al. 2023; Power et al. 2012). Although the greater percentage of missing data in more stringent pathways might exacerbate concerns about missing data handling, biases in additional exclusions as QC became more stringent were only modestly stronger than earlier exclusions—that is, adopting a lenient motion scrubbing threshold does not yield a bias‐free sample. On the other hand, we would not recommend the adoption of QC procedures which are unnecessarily stringent. Our analysis showed that a scrubbing threshold of 0.1 mm, for example, excluded a much greater proportion of the data than a threshold of 0.2 mm without improving our metric of motion artifacts. Relatedly, Kay et al. (2023) found that censoring at 0.1 mm does not reduce false positives from motion artifact compared to 0.2 mm scrubbing in ABCD. Exclusions are even greater at thresholds under 0.1 mm—as a well‐known example, Marek et al. (2022) primary analysis thresholded at 0.08 mm, yielding a sample of only 3,928 (sensitivity tests were performed in a larger subsample). Our finding that these exclusions are not completely at random suggests a substantial cost in accuracy to approaches filtering at motion thresholds under 0.2 mm, apparently with little improvement in noise filtering. Instead, we recommend handling missing data analytically (as allowable), interpreting findings in light of methodological limitations, and improving practices in neuroimaging to reduce the need for data exclusion.

### 3.2 Recommendations for Improved Practices in Research Design and Methodology

#### 3.2.1 Investigate the Causes of Motion

Improved practices are needed to increase the quality of acquired data, especially in pediatric neuroimaging. Associations between motion and participant characteristics and motion should be investigated. For example, BMI was related to exclusion after motion thresholding—why is this? Are higher BMI individuals less comfortable in the scanner, and/or are they positioned in the head coil in such a way as to make motion more likely? How could scanner equipment be adapted to better accommodate higher BMI participants? Similarly, how can the scanning session be designed to minimize discomfort for neurodivergent individuals or those with psychopathology and/or trauma histories? We have demonstrated that these are urgent methodological questions.

#### 3.2.2 Improve Practices in Data Acquisition and Pre‐Processing

Secondly, there is a need for improved methods during data acquisition and analysis to correct for data quality artifacts and missing data. Real time monitoring of subject motion (e.g., using tools such as real‐time AFNI or FIRMM) could allow interventions during acquisition to reduce motion. These could include coaching the participant, adjusting subject position or head padding, or acquiring additional compensatory data (e.g., Smith et al. 2022); ABCD employed FIRMM, but only at sites with Siemens scanners (Casey et al. 2018). Prospective motion correction (PMC) during acquisition may improve rs‐fMRI data quality in participants exhibiting motion (Hoinkiss et al. 2019; Maziero et al. 2020; Zaitsev et al. 2017), and further improvements in PMC methodology may substantially decrease the impact of motion on data quality. Acquisition methods like multi‐echo fMRI can allow for improved motion correction and denoising (Kundu et al. 2017). Motion impacts on missingness may be further reduced by increasing resting state scanner time and/or supplementing resting state analyses with task based fMRI data (although this latter method may modulate FC; Frank and Zeithamova 2023), thereby increasing the amount of data that can be censored without excluding participants. Playing a movie during resting state acquisition may reduce head motion (especially in children under 12), but also may modulate functional connectivity (Vanderwal, Eilbott, and Castellanos 2018). The impact of motion on trait‐FC correlations can be quantified and reported on to enhance the interpretability of findings that may be affected by motion (Kay et al. 2023). Motion‐ordering and resampling (bagging) may allow analysts to safely retain higher motion participants during analysis while simultaneously limiting the impact of motion on identified brain‐behavior relationships (Ramduny et al. 2024). This would not only increase the analysis sample size, but also increase representativeness of the final sample. Finally, implementation of formal missing data handling in large neuroimaging datasets is computationally and practically challenging. New tools and expert guidance for applying methods such as multiple imputation and FIML to such datasets could increase their adoption by researchers.

Given the sensitivity of our results to site, we recommend special care be taken in analysis of multi‐site data. Because sites' recruitment areas have different demographics, differences in practices across sites can easily result in a biased pattern of exclusions. Analysts should be vigilant for processing steps which disproportionately remove cases from specific sites. During analysis, statistical control may help detect these biases but could also obscure effects of relevant sociodemographic variables. When site effects exist, it is recommended that results be analyzed both with and without control for site.

#### 3.2.3 Improve Transparency and Replicability of Quality Control Procedures

Finally, a third set of improvements concerns improvements in transparency and replicability. Information about the reasoning behind inclusion recommendations in ABCD is frequently unclear. For example, some images were not present in the ABCD release v4.0 tabulated data because they “failed visual inspection,” but to our knowledge no protocol has been published about what sorts of anomalies were considered unusable. In the course of our investigation, we discovered that subject inclusion recommendations varied considerably between different releases of the ABCD dataset, and that this variation led to major differences in subject lists between curated data releases (i.e., between ABCC and ABCD release 4). Because earlier versions of the dataset were not always available (e.g., earlier versions of the fast‐track data operator QC report), these different subject lists were not reproducible. Consequently, analyses based on those datasets are no longer reproducible from publicly available inputs. Information about data quality (QC recommendations, including notes) are a critical component of analysis in all neuroimaging research and should be considered part of the formal dataset—this is especially true given widespread variability in how research teams perform QC and the specificity of QC decisions to research questions (Taylor et al. 2023). To preserve the open‐ness of open datasets, the protocols, computer code, and data used to make QC recommendations, as well as the recommendations themselves, should be publicly available with adequate version control to allow reproducibility of analyses after the data has been revised. Without this information, it is difficult to ascertain how quality issues in the dataset might be expected to affect publications which were based on previous dataset versions. Relatedly, a 2024 review found that a minority of studies currently published using ABCD data followed best‐practice recommendations to report characteristics of excluded participants, quantify missing data on a variable level, discuss missing data mechanisms, and use imputation methods to account for missingness (Lopez et al. 2024). We recommend that analysts using ABCD and similar datasets adopt these practices. Adoption would be supported by better documentation of quality control procedures and recommendations in future ABCD releases.

### 3.3 Limitations

Our investigation had a number of limitations. First, our metrics of motion bias in the data are limited: while a sloped QC‐FC plot is indicative of some amount of motion bias in the data, a flat plot is not sufficient to prove there is no motion bias in the data. Second, the relation of race/ethnicity and neighborhood characteristics to missingness appeared to be sensitive to control for scanner site—this reflect higher rates of exclusion in ABCC from some sites vs. ABCD release v4.0, which may be correctable in future releases of ABCC and thus may not affect future work based on said future releases. We employed a p‐factor as an index of psychopathology—the interpretability of this approach has been contested. However, the p‐factor appears to be an effective index of total psychopathology severity (Watts et al. 2024), and its orthogonality to the internalizing and externalizing subfactors is helpful for this analysis. We only examined missingness of entire cases—an additional question meriting further study is whether frame‐wise censoring obscures important individual variation in functional connectivity (i.e., is there something happening in the brain during censored frames which contains important information about included individuals' over‐all pattern of functional connectivity). We examined ABCD release v4.0, which has since been superseded by v5.0—newer releases of ABCD contain more granular descriptions of image quality, but DAIRC's recommended inclusion list for rs‐fMRI data is largely unchanged. We examined only baseline data and are unable to comment on changes in exclusion patterns over time. Lastly, we were able to test whether rs‐fMRI data was contingent on non‐imaging variables, but not whether it was missing contingent on brain connectivity (i.e., MNAR). Whether the data is MNAR is an important consideration when planning analyses but cannot be determined empirically.

### 3.4 Conclusion

We demonstrated that a wide variety of participant characteristics in the ABCD dataset are systematically related to QC‐related exclusions of rs‐fMRI data, and that this pattern holds at all reasonable levels of motion scrubbing and in two separate QC processing streams, extending previous work on this topic (Cosgrove et al. 2022). Our results suggest that formal handling of missing data is necessary when examining rs‐fMRI data in large datasets such as ABCD. Furthermore, even given such handling, results should be interpreted cautiously given the strong possibility that exclusions are contingent on brain FC and thus biased. Additionally, these findings suggest that efforts to reduce QC related exclusion of rs‐fMRI data are important to ensure the accuracy and generalizability of findings resulting from rs‐fMRI studies in the future, especially including those at high risk of exclusion such as children. Finally, our results underscore the importance of transparency and availability of data quality related information in open datasets such as ABCD.

## Author Contributions

Conceptualization: M.P.; data curation: M.P. and J.D.R.; formal analysis: M.P.; funding acquisition: M.P., R.M.B. and R.J.H.; methodology: M.P., M.A.H. and K.M.K.; project administration: M.P.; resources: R.M.B. and R.J.H.; software: M.P., T.J.K.; supervision: J.D.R., R.M.B. and R.J.H.; visualization: M.P., T.J.K. and H.M.R.; writing – original draft: M.P. and T.J.K.; writing – review and editing: M.P., J.D.R., T.J.K., H.M.R., M.A.H., K.M.K., R.M.B. and R.J.H.

## Conflicts of Interest

R.M.B. has consulted with Turing Medical on the development of FIRMM. R.J.H. has served as a consultant for Jazz Pharmaceuticals. No other authors have conflicts of interest to declare.

## Supporting information


Data S1.

## Data Availability

All data analyzed is publicly available via the National Data Archive (https://nda.nih.gov/). All analysis code is available on osf (https://osf.io/57xer/) and in our Supporting Information.

## References

[hbm70094-bib-0001] Achenbach, T. M. , L. Dumenci , and L. A. Rescorla . 2002. “Ten‐Year Comparisons of Problems and Competencies for National Samples of Youth: Self, Parent, and Teacher Reports.” Journal of Emotional and Behavioral Disorders 10, no. 4: 194–203. 10.1177/10634266020100040101.

[hbm70094-bib-0002] Akshoomoff, N. , J. L. Beaumont , P. J. Bauer , et al. 2013. “Nih Toolbox Cognition Battery (Cb): Composite Scores of Crystallized, Fluid, and Overall Cognition.” Monographs of the Society for Research in Child Development 78, no. 4: 119–132. 10.1111/mono.12038. 23952206 PMC4103789

[hbm70094-bib-0003] Baraldi, A. N. , and C. K. Enders . 2010. “An Introduction to Modern Missing Data Analyses.” Journal of School Psychology 48, no. 1: 5–37. 10.1016/j.jsp.2009.10.001. 20006986

[hbm70094-bib-0004] Behzadi, Y. , K. Restom , J. Liau , and T. T. Liu . 2007. “A Component Based Noise Correction Method (CompCor) for BOLD and Perfusion Based fMRI.” NeuroImage 37, no. 1: 90–101. 10.1016/j.neuroimage.2007.04.042. 17560126 PMC2214855

[hbm70094-bib-0005] Brislin, S. J. , M. E. Martz , S. Joshi , et al. 2021. “Differentiated Nomological Networks of Internalizing, Externalizing, and the General Factor of Psychopathology ('p Factor’) in Emerging Adolescence in the ABCD Study.” Psychological Medicine 1–11: 3051–3061. 10.1017/S0033291720005103. PMC9693677 33441214

[hbm70094-bib-0006] Casey, B. J. , T. Cannonier , M. I. Conley , et al. 2018. “The Adolescent Brain Cognitive Development (ABCD) Study: Imaging Acquisition Across 21 Sites.” Developmental Cognitive Neuroscience 32: 43–54. 10.1016/j.dcn.2018.03.001. 29567376 PMC5999559

[hbm70094-bib-0007] Cheng, T. W. , L. Magis‐Weinberg , V. Guazzelli Williamson , et al. 2021. “A Researcher's Guide to the Measurement and Modeling of Puberty in the ABCD Study® at Baseline.” Frontiers in Endocrinology 12: 1–9. 10.3389/fendo.2021.608575. PMC8131843 34025573

[hbm70094-bib-0008] Ciric, R. , A. F. G. Rosen , G. Erus , et al. 2018. “Mitigating Head Motion Artifact in Functional Connectivity MRI.” Nature Protocols 13, no. 12: 2801–2826. 10.1038/s41596-018-0065-y. 30446748 PMC8161527

[hbm70094-bib-0009] Ciric, R. , D. H. Wolf , J. D. Power , et al. 2017. “Benchmarking of Participant‐Level Confound Regression Strategies for the Control of Motion Artifact in Studies of Functional Connectivity.” NeuroImage 154: 174–187. 10.1016/j.neuroimage.2017.03.020. 28302591 PMC5483393

[hbm70094-bib-0010] Clark, D. A. , B. M. Hicks , M. Angstadt , et al. 2021. “The General Factor of Psychopathology in the Adolescent Brain Cognitive Development (ABCD) Study: A Comparison of Alternative Modeling Approaches.” Clinical Psychological Science: A Journal of the Association for Psychological Science 9, no. 2: 169–182. 10.1177/2167702620959317. 34621600 PMC8494184

[hbm70094-bib-0011] Clark, D. B. , C. B. Fisher , S. Bookheimer , et al. 2018. “Biomedical Ethics and Clinical Oversight in Multisite Observational Neuroimaging Studies With Children and Adolescents: The ABCD Experience.” Developmental Cognitive Neuroscience 32: 143–154. 10.1016/j.dcn.2017.06.005. 28716389 PMC5745294

[hbm70094-bib-0012] Cosgrove, K. T. , T. J. McDermott , E. J. White , et al. 2022. “Limits to the Generalizability of Resting‐State Functional Magnetic Resonance Imaging Studies of Youth: An Examination of ABCD Study® Baseline Data.” Brain Imaging and Behavior 16, no. 4: 1919–1925. 10.1007/s11682-022-00665-2. 35552993 PMC9296258

[hbm70094-bib-0013] Dotson, V. M. , and A. Duarte . 2020. “The Importance of Diversity in Cognitive Neuroscience.” Annals of the New York Academy of Sciences 1464, no. 1: 181–191. 10.1111/nyas.14268. 31663150

[hbm70094-bib-0014] Fadus, M. C. , E. A. Valadez , B. E. Bryant , et al. 2021. “Racial Disparities in Elementary School Disciplinary Actions: Findings From the ABCD Study.” Journal of the American Academy of Child & Adolescent Psychiatry 60, no. 8: 998–1009. 10.1016/j.jaac.2020.11.017. 33359407 PMC8860403

[hbm70094-bib-0015] Fair, D. A. , O. Miranda‐Dominguez , A. Z. Snyder , et al. 2020. “Correction of Respiratory Artifacts in MRI Head Motion Estimates.” NeuroImage 208: 116400. 10.1016/j.neuroimage.2019.116400. 31778819 PMC7307712

[hbm70094-bib-0016] Fakkel, M. , M. Peeters , P. Lugtig , et al. 2020. “Testing Sampling Bias in Estimates of Adolescent Social Competence and Behavioral Control.” Developmental Cognitive Neuroscience 46: 100872. 10.1016/j.dcn.2020.100872. 33142133 PMC7642800

[hbm70094-bib-0017] Fan, C. C. , A. Marshall , H. Smolker , et al. 2021. “Adolescent Brain Cognitive Development (ABCD) Study Linked External Data (LED): Protocol and Practices for Geocoding and Assignment of Environmental Data.” Developmental Cognitive Neuroscience 52: 101030. 10.1016/j.dcn.2021.101030. 34891080 PMC8666341

[hbm70094-bib-0018] Feczko, E. , G. Conan , S. Marek , et al. 2021. “Adolescent Brain Cognitive Development (ABCD) Community MRI Collection and Utilities.” bioRxiv, 2021.07.09.451638. 10.1101/2021.07.09.451638.

[hbm70094-bib-0019] Finn, E. S. , X. Shen , D. Scheinost , et al. 2015. “Functional Connectome Fingerprinting: Identifying Individuals Based on Patterns of Brain Connectivity.” Nature Neuroscience 18, no. 11: 1664–1671. 10.1038/nn.4135. 26457551 PMC5008686

[hbm70094-bib-0020] Frank, L. E. , and D. Zeithamova . 2023. “Evaluating Methods for Measuring Background Connectivity in Slow Event‐Related Functional Magnetic Resonance Imaging Designs.” Brain and Behavior: A Cognitive Neuroscience Perspective 13, no. 6: e3015. 10.1002/brb3.3015. PMC10275534 37062880

[hbm70094-bib-0021] Frew, S. , A. Samara , H. Shearer , J. Eilbott , and T. Vanderwal . 2022. “Getting the Nod: Pediatric Head Motion in a Transdiagnostic Sample During Movie‐ and Resting‐State fMRI.” PLoS One 17, no. 4: e0265112. 10.1371/journal.pone.0265112. 35421115 PMC9009630

[hbm70094-bib-0022] Garavan, H. , H. Bartsch , K. Conway , et al. 2018. “Recruiting the ABCD Sample: Design Considerations and Procedures.” Developmental Cognitive Neuroscience 32: 16–22. 10.1016/j.dcn.2018.04.004. 29703560 PMC6314286

[hbm70094-bib-0023] Gard, A. M. , L. W. Hyde , S. G. Heeringa , B. T. West , and C. Mitchell . 2023. “Why Weight? Analytic Approaches for Large‐Scale Population Neuroscience Data.” Developmental Cognitive Neuroscience 59: 101196. 10.1016/j.dcn.2023.101196. 36630774 PMC9843279

[hbm70094-bib-0024] Gelman, A. , and E. Loken . 2013. “The Garden of Forking Paths: Why Multiple Comparisons Can Be a Problem, Even When There Is no “Fishing Expedition” or “p‐Hacking” and the Research Hypothesis Was Posited Ahead of Time.” https://stat.columbia.edu/~gelman/research/unpublished/p_hacking.pdf.

[hbm70094-bib-0025] Glasser, M. F. , T. S. Coalson , E. C. Robinson , et al. 2016. “A Multi‐Modal Parcellation of Human Cerebral Cortex.” Nature 536, no. 7615: 171–178. 10.1038/nature18933. 27437579 PMC4990127

[hbm70094-bib-0026] Green, K. H. , I. H. Van De Groep , L. W. Te Brinke , R. van der Cruijsen , F. van Rossenberg , and H. El Marroun . 2022. “A Perspective on Enhancing Representative Samples in Developmental Human Neuroscience: Connecting Science to Society.” Frontiers in Integrative Neuroscience 16: 981657. 10.3389/fnint.2022.981657. 36118120 PMC9480848

[hbm70094-bib-0027] Hagler, D. J. , S. N. Hatton , M. D. Cornejo , et al. 2019. “Image Processing and Analysis Methods for the Adolescent Brain Cognitive Development Study.” NeuroImage 202: 116091. 10.1016/j.neuroimage.2019.116091. 31415884 PMC6981278

[hbm70094-bib-0028] Harrell, F. E. 2015. “Regression Modeling Strategies: With Applications to Linear Models, Logistic and Ordinal Regression, and Survival Analysis.” Springer International Publishing. 10.1007/978-3-319-19425-7.

[hbm70094-bib-0029] Hausman, H. K. , C. Hardcastle , J. N. Kraft , et al. 2022. “The Association Between Head Motion During Functional Magnetic Resonance Imaging and Executive Functioning in Older Adults.” Neuroimage: Reports 2, no. 2: 100085. 10.1016/j.ynirp.2022.100085. 37377763 PMC10299743

[hbm70094-bib-0030] Heeringa, S. G. , and P. A. Berglund . 2020. “A Guide for Population‐Based Analysis of the Adolescent Brain Cognitive Development (ABCD) Study Baseline Data.” bioRxiv, 2020.02.10.942011. 10.1101/2020.02.10.942011.

[hbm70094-bib-0031] Hodgson, K. , R. A. Poldrack , J. E. Curran , et al. 2017. “Shared Genetic Factors Influence Head Motion During MRI and Body Mass Index.” Cerebral Cortex 27, no. 12: 5539–5546. 10.1093/cercor/bhw321. 27744290 PMC6075600

[hbm70094-bib-0032] Hoinkiss, D. C. , P. Erhard , N.‐J. Breutigam , F. von Samson‐Himmelstjerna , M. Günther , and D. A. Porter . 2019. “Prospective Motion Correction in Functional MRI Using Simultaneous Multislice Imaging and Multislice‐To‐Volume Image Registration.” NeuroImage 200: 159–173. 10.1016/j.neuroimage.2019.06.042. 31226496

[hbm70094-bib-0033] Iacono, W. G. , A. C. Heath , J. K. Hewitt , et al. 2018. “The Utility of Twins in Developmental Cognitive Neuroscience Research: How Twins Strengthen the ABCD Research Design.” Developmental Cognitive Neuroscience 32: 30–42. 10.1016/j.dcn.2017.09.001. 29107609 PMC5847422

[hbm70094-bib-0034] Jernigan, T. L. , S. A. Brown , A. M. Dale , et al. 2021. “*Adolescent Brain Cognitive Development Study (ABCD)—Annual Release 4.0*.” 10.15154/1523041.

[hbm70094-bib-0035] Kay, B. P. , D. F. Montez , S. Marek , et al. 2023. “Motion Impact Score for Detecting Spurious Brain‐Behavior Associations.” bioRxiv, 2022.12.16.520797. 10.1101/2022.12.16.520797. PMC12479937 41022827

[hbm70094-bib-0036] Kundu, P. , V. Voon , P. Balchandani , M. V. Lombardo , B. A. Poser , and P. A. Bandettini . 2017. “Multi‐Echo fMRI: A Review of Applications in fMRI Denoising and Analysis of BOLD Signals.” NeuroImage 154: 59–80. 10.1016/j.neuroimage.2017.03.033. 28363836

[hbm70094-bib-0037] Lee, T. , and D. Shi . 2021. “A Comparison of Full Information Maximum Likelihood and Multiple Imputation in Structural Equation Modeling With Missing Data.” Psychological Methods 26, no. 4: 466–485. 10.1037/met0000381. 33507765

[hbm70094-bib-0038] Lopez, D. A. , C. Cardenas‐Iniguez , P. Subramaniam , et al. 2024. “Transparency and Reproducibility in the Adolescent Brain Cognitive Development (ABCD) Study.” Developmental Cognitive Neuroscience 68: 101408. 10.1016/j.dcn.2024.101408. 38924835 PMC11254940

[hbm70094-bib-0039] Marek, S. , B. Tervo‐Clemmens , F. J. Calabro , et al. 2022. “Reproducible Brain‐Wide Association Studies Require Thousands of Individuals.” Nature 603, no. 7902: 654–660. 10.1038/s41586-022-04492-9. 35296861 PMC8991999

[hbm70094-bib-0040] Maziero, D. , C. Rondinoni , T. Marins , V. A. Stenger , and T. Ernst . 2020. “Prospective Motion Correction of fMRI: Improving the Quality of Resting State Data Affected by Large Head Motion.” NeuroImage 212: 116594. 10.1016/j.neuroimage.2020.116594. 32044436 PMC7238750

[hbm70094-bib-0041] McNeilly, E. A. , M. Peverill , J. Jung , and K. A. McLaughlin . 2021. “Executive Function as a Mechanism Linking Socioeconomic Status to Internalizing and Externalizing Psychopathology in Children and Adolescents.” Journal of Adolescence 89: 149–160. 10.1016/j.adolescence.2021.04.010. 33971502 PMC8203104

[hbm70094-bib-0042] Myatt, M. , and E. Guevarra . 2019. “Zscorer: Child Anthropometry z‐Score Calculator.” https://CRAN.R‐project.org/package=zscorer.

[hbm70094-bib-0043] Noble, K. G. , B. D. McCandliss , and M. J. Farah . 2007. “Socioeconomic gradients predict individual differences in neurocognitive abilities.” Developmental Science 10, no. 4: 464–480. 10.1111/j.1467-7687.2007.00600.x. 17552936

[hbm70094-bib-0044] Parkes, L. , B. Fulcher , M. Yücel , and A. Fornito . 2018. “An Evaluation of the Efficacy, Reliability, and Sensitivity of Motion Correction Strategies for Resting‐State Functional MRI.” NeuroImage 171: 415–436. 10.1016/j.neuroimage.2017.12.073. 29278773

[hbm70094-bib-0045] Petersen, A. C. , L. Crockett , M. Richards , and A. Boxer . 1988. “A Self‐Report Measure of Pubertal Status: Reliability, Validity, and Initial Norms.” Journal of Youth and Adolescence 17, no. 2: 117–133. 10.1007/BF01537962. 24277579

[hbm70094-bib-0046] Power, J. D. , K. A. Barnes , A. Z. Snyder , B. L. Schlaggar , and S. E. Petersen . 2012. “Spurious but Systematic Correlations in Functional Connectivity MRI Networks Arise From Subject Motion.” NeuroImage 59, no. 3: 2142–2154. 10.1016/j.neuroimage.2011.10.018. 22019881 PMC3254728

[hbm70094-bib-0047] Power, J. D. , A. Mitra , T. O. Laumann , A. Z. Snyder , B. L. Schlaggar , and S. E. Petersen . 2014. “Methods to Detect, Characterize, and Remove Motion Artifact in Resting State fMRI.” NeuroImage 84: 320–341. 10.1016/j.neuroimage.2013.08.048. 23994314 PMC3849338

[hbm70094-bib-0048] Power, J. D. , B. L. Schlaggar , and S. E. Petersen . 2015. “Recent Progress and Outstanding Issues in Motion Correction in Resting State fMRI.” NeuroImage 105: 536–551. 10.1016/j.neuroimage.2014.10.044. 25462692 PMC4262543

[hbm70094-bib-0049] Pruim, R. H. R. , M. Mennes , D. van Rooij , A. Llera , J. K. Buitelaar , and C. F. Beckmann . 2015. “ICA‐AROMA: A Robust ICA‐Based Strategy for Removing Motion Artifacts From fMRI Data.” NeuroImage 112: 267–277. 10.1016/j.neuroimage.2015.02.064. 25770991

[hbm70094-bib-0050] Ramduny, J. , L. Q. Uddin , T. Vanderwal , et al. 2024. “Increasing the Representation of Minoritized Youth for Inclusive and Reproducible Brain‐Behavior Association.” bioRxiv, 2024.06.22.600221. 10.1101/2024.06.22.600221.

[hbm70094-bib-0051] Rhoades, B. L. , M. T. Greenberg , S. T. Lanza , and C. Blair . 2011. “Demographic and Familial Predictors of Early Executive Function Development: Contribution of a Person‐Centered Perspective.” Journal of Experimental Child Psychology 108, no. 3: 638–662. 10.1016/j.jecp.2010.08.004. 20828709 PMC3016464

[hbm70094-bib-0052] Ricard, J. A. , T. C. Parker , E. Dhamala , J. Kwasa , A. Allsop , and A. J. Holmes . 2023. “Confronting Racially Exclusionary Practices in the Acquisition and Analyses of Neuroimaging Data.” Nature Neuroscience 26, no. 1: 4–11. 36564545 10.1038/s41593-022-01218-y PMC12884511

[hbm70094-bib-0053] Saragosa‐Harris, N. M. , N. Chaku , N. MacSweeney , et al. 2022. “A Practical Guide for Researchers and Reviewers Using the ABCD Study and Other Large Longitudinal Datasets.” Developmental Cognitive Neuroscience 55: 101115. 10.1016/j.dcn.2022.101115. 35636343 PMC9156875

[hbm70094-bib-0054] Satterthwaite, T. D. , M. A. Elliott , R. T. Gerraty , et al. 2013. “An Improved Framework for Confound Regression and Filtering for Control of Motion Artifact in the Preprocessing of Resting‐State Functional Connectivity Data.” NeuroImage 64: 240–256. 10.1016/j.neuroimage.2012.08.052. 22926292 PMC3811142

[hbm70094-bib-0055] Satterthwaite, T. D. , D. H. Wolf , J. Loughead , et al. 2012. “Impact of In‐Scanner Head Motion on Multiple Measures of Functional Connectivity: Relevance for Studies of Neurodevelopment in Youth.” NeuroImage 60, no. 1: 623–632. 10.1016/j.neuroimage.2011.12.063. 22233733 PMC3746318

[hbm70094-bib-0056] Singh, G. K. 2003. “Area Deprivation and Widening Inequalities in US Mortality, 1969–1998.” American Journal of Public Health 93, no. 7: 1137–1143. 10.2105/AJPH.93.7.1137. 12835199 PMC1447923

[hbm70094-bib-0057] Smith, J. , E. Wilkey , B. Clarke , et al. 2022. “Can This Data Be Saved? Techniques for High Motion in Resting State Scans of First Grade Children.” Developmental Cognitive Neuroscience 58: 101178. 10.1016/j.dcn.2022.101178. 36434964 PMC9694086

[hbm70094-bib-0058] Taylor, P. A. , D. R. Glen , R. C. Reynolds , A. Basavaraj , D. Moraczewski , and J. A. Etzel . 2023. “Editorial: Demonstrating Quality Control (QC) Procedures in fMRI.” Frontiers in Neuroscience 17: 1–11. 10.3389/fnins.2023.1205928. PMC10264898 37325035

[hbm70094-bib-0059] Thompson, W. K. , D. M. Barch , J. M. Bjork , et al. 2019. “The Structure of Cognition in 9 and 10 Year‐Old Children and Associations With Problem Behaviors: Findings From the ABCD study's Baseline Neurocognitive Battery.” Developmental Cognitive Neuroscience 36: 100606. 10.1016/j.dcn.2018.12.004. 30595399 PMC6676481

[hbm70094-bib-0060] Townsend, L. , K. Kobak , C. Kearney , et al. 2020. “Development of Three Web‐Based Computerized Versions of the Kiddie Schedule for Affective Disorders and Schizophrenia Child Psychiatric Diagnostic Interview: Preliminary Validity Data.” Journal of the American Academy of Child and Adolescent Psychiatry 59, no. 2: 309–325. 10.1016/j.jaac.2019.05.009. 31108163

[hbm70094-bib-0061] Van Dijk, K. R. A. , T. Hedden , A. Venkataraman , K. C. Evans , S. W. Lazar , and R. L. Buckner . 2010. “Intrinsic Functional Connectivity as a Tool for Human Connectomics: Theory, Properties, and Optimization.” Journal of Neurophysiology 103, no. 1: 297–321. 10.1152/jn.00783.2009. 19889849 PMC2807224

[hbm70094-bib-0062] Vanderwal, T. , J. Eilbott , and F. X. Castellanos . 2018. “Movies in the Magnet: Naturalistic Paradigms in Developmental Functional Neuroimaging.” Developmental Cognitive Neuroscience 36: 100600. 10.1016/j.dcn.2018.10.004. 30551970 PMC6969259

[hbm70094-bib-0063] Watts, A. L. , A. L. Greene , W. Bonifay , and E. I. Fried . 2024. “A Critical Evaluation of the p‐Factor Literature.” Nature Reviews Psychology 3, no. 2: 108–122. 10.1038/s44159-023-00260-2.

[hbm70094-bib-0064] Woods, A. D. , D. Gerasimova , B. Van Dusen , et al. 2023. “Best Practices for Addressing Missing Data Through Multiple Imputation.” Infant and Child Development 33, no. 1: e2407. 10.1002/icd.2407.

[hbm70094-bib-0065] Zaitsev, M. , B. Akin , P. LeVan , and B. R. Knowles . 2017. “Prospective Motion Correction in Functional MRI.” NeuroImage 154: 33–42. 10.1016/j.neuroimage.2016.11.014. 27845256 PMC5427003

