# Supplementary material for: Balancing Data Quality and Bias: Investigating Functional Connectivity Exclusions in the Adolescent Brain Cognitive Development℠ (ABCD Study) Across Quality Control Pathways
Source: Hum Brain Mapp. 2025 Jan 9;46(1):e70094. doi: 10.1002/hbm.70094 (PMC11717557; doi:10.1002/hbm.70094)
Supplement: Supplementary file 1 — Data S1. [file HBM-46-e70094-s001.pdf]

Balancing data quality and bias: Investigating functional connectivity exclusions in the Adolescent Brain Cognitive Development^{SM} (ABCD Study®) across quality control pathways

Supplemental Methods and Results

Matthew Peverill, Justin D. Russell, Taylor J. Keding, Hailey M. Rich, Max A. Halvorson, Kevin M. King, Rasmus M. Birn, & Ryan J. Herringa

2024-12-09

Contents

1 Supplemental Methods **3**
1.1 Behavioral and Demographic Variables Detailed Information. 3
1.1.1 Neighborhood Factors 3
1.1.2 Trauma and Stress Exposure. 3
1.1.3 Participant Demographics. 4
1.1.4 Pubertal Development. 4
1.1.5 Psychopathology. 4
1.1.6 General Cognitive Ability. 4
1.1.7 Inhibitory Control. 4
1.1.8 Body Mass Index. 4

2 Code Supplement **5**

3 Supplementary Results **5**
3.1 F-tests for discrete variable-Motion associations 5
3.2 Relation of participant characteristics to scan time (post scrubbing) 5
3.3 Multiply at Risk Cell Counts 5
3.4 Categorical Variable Table by Condition 5
3.5 Missingness in Behavioral Data 5

3.6 Adjusted Models – Forest Plot . . . 5
3.7 Sensitivity to Site/Scanner . . . 5
3.7.1 Sensitivity to Site/Scanner Table . . . 12
3.7.2 Sensitivity to Site/Scanner Forest Plots . . . 12
3.8 Current Fast Track QC vs DAIRC recommendations . . . 12
3.9 Modeling Trends Across Conditions (H2) . . . 12
3.9.1 H2 Marginal Means plotting (Figure 5) . . . 16
3.10 Association of QC missingness with Propensity weighting. . . . 16
3.11 Association of Exclusion with Expanded Demographic Variables . . . 19
3.11.1 Household Income . . . 19
3.11.2 Detailed Race/Ethnicity . . . 19

4 Changes since pre-registration **25**
4.1 Save Tables . . . 25

5 Supplemental References **25**

6 SessionInfo **34**

List of Tables

1 F tests for relation of discrete DVs with FD after scrubbing, by threshold (pt. 1) . . . 6
2 F tests for relation of discrete DVs with FD after scrubbing, by threshold (pt. 2) . . . 7
3 Non-white (census) with psychopathology at z >= 1.5 . . . 7
4 Male participants with NIH toolbox total scores of z <= -1.5 . . . 7
5 Categorical Values at Each Level of Stringency (Subjects excluded with < 375 Frames), Pt 1 . . . 8
6 Behavioral and Demographic variables with >50 missing values . . . 9
7 Sites with Highest % Excluded, by Condition . . . 9
8 OR of scanner type effect, with and without site control, in condition adjusted models [95% CI]. ***: p<.001 . . . 12
9 FastQC vs Tabulated Data Inclusion/Exclusion . . . 13
10 FastQC vs DAIRC recommended Inclusion/Exclusion . . . 13
11 Comparison of Model Performance Indices . . . 15

12 H2 / Omnibus model fixed effects, pt. 1 . . . 17
13 H2 / Omnibus model fixed effects, pt. 2 . . . 18
14 Propensity weights of included vs. excluded participants by condition; t-test parameters . . . 23
15 Granular Household Income at Each Level of Stringency (Subjects excluded with < 375 Frames) . . . 23
16 Bivariate Model of Granular Household Income and Missingness by Condition. Ref: $100k-$199k . . . 24
17 Percentage Endorsing Granular Race/Ethnicity Variables within Census Race/Ethnicity Groups . . . 26
18 Granular Race/Ethnicity Data by Condition . . . 27
19 Granular Race/Ethnicity Variables: Bivariate Models, Pt. 1 . . . 28
20 Granular Race/Ethnicity Variables: Bivariate Models, Pt. 2 . . . 29
21 Granular Race/Ethnicity Variables: Bivariate Models, Pt. 3 . . . 30
22 Adjusted Models Output, as Pre-registered (Pt. 1). ***: p<.001; **: p<.01; *:p<.05 . . . 31
23 Adjusted Models Output, as Pre-registered (Pt. 2). ***: p<.001; **: p<.01; *:p<.05 . . . 32

1 Supplemental Methods

1.1 Behavioral and Demographic Variables Detailed Information.

1.1.1 Neighborhood Factors

Area Deprivation Index (ADI) is a composite measure of a neighborhood's relative socioeconomic disadvantage as compared to other US neighborhoods (Singh, 2003). ADI has shown interactive effects with individual SES measures in predicting rs-fMRI functional connectivity in the ABCD dataset (Rakesh et al., 2021). The Child Opportunity Index (v2.0; COI2) is a composite index derived from census data that indexes neighborhood resources and conditions relevant to children's development, inclusive of educational opportunities, health-affecting and environmental factors, and social and economic resources (Fan et al., 2021). When participants listed multiple residences, averages of these neighborhood factors were calculated and weighted according to time in residence.

1.1.2 Trauma and Stress Exposure.

Youth exposure to DSM-5 PTSD criterion-A eligible traumatic experiences, as measured by the parent-report Kiddie Schedule for Affective Disorders and Schizophrenia (KSADS; Townsend et al., 2020), was used as an index of traumatic stress exposure. We coded trauma exposure categorically as unexposed, single exposure, or multiple (two or more) exposures. This approach, when employed with a nationally representative sample of adolescents aged 13-17, roughly divided sample into tertiles for exposure severity (McLaughlin et al., 2013). This approach allows for specific examination of missingness among multiply exposed youth, who show especially high risk for PTSD (McLaughlin et al., 2013) and other adverse outcomes (Evans & Kim, 2010), and does so without dividing the sample into undersized sub-groups.

1.1.3 Participant Demographics.

Participant demographic variables included total family income, highest parental education, race/ethnicity, and sex assigned at birth. Income was re-leveled from ten income ranges to eight to allow for larger subject counts within each range (see Heeringa & Berglund, 2020). Caregiver education was similarly re-leveled from twenty-one levels to five; we expected minimal variation in exclusions within these five bins given findings from previous work with ABCD (Cosgrove et al., 2022). We used U.S. Census derived race/ethnicity categories included in ABCD to facilitate comparisons with existing literature (e.g., Cosgrove et al., 2022; Fadus et al., 2021; Heeringa & Berglund, 2020); notably, however, this approach may under-represent important U.S. sub-populations (Saragosa-Harris et al., 2022). Secondary analyses using the fully expanded variables are included in supplemental materials.

1.1.4 Pubertal Development.

We include pubertal development category scores (pre/early/mid/late/post-pubertal) derived from the Pubertal Development Scale (PDS; Petersen et al., 1988). We used the caregiver reported scores, as they may be more accurate in the age range (9-10 years) of the ABCD baseline sample (Cheng et al., 2021), but substituted child reported scores where caregiver reports were missing.

1.1.5 Psychopathology.

In order to separately examine the impact of general, internalizing, and externalizing psychopathology, we calculated a generalized psychopathology factor (P), as well as residual internalizing and externalizing factors, by fitting a bifactor model to the 8 subscales of the Child Behavior Checklist (CBCL; Achenbach et al., 2002; Brislin et al., 2021; D. A. Clark et al., 2021). The model showed acceptable fit to the data (^{2} = 1338.249, df = 15, $p < 0.001$, RMSEA = 0.086, CFI = 0.973, TLI = 0.949, SRMR = 0.028). P-factor scores derived from the model were strongly correlated (r=.87) with the Total Problems subscale of the CBCL.

1.1.6 General Cognitive Ability.

Cognitive ability was operationalized using the matrix reasoning task scaled score from the WISC-V as well as the cognition composite and crystalized intelligence composite scores captured by the NIH Toolbox (Akshoomoff et al., 2013; Thompson et al., 2019). Extant research affirms the reliability and validity of each measure in youth samples (Akshoomoff et al., 2013; Canivez et al., 2020; Thompson et al., 2019).

1.1.7 Inhibitory Control.

The NIH Toolbox Flanker Inhibitory Control task score (Weintraub et al., 2013) was included as a behavioral measure of inhibitory control. Scores on this task have been correlated with both parent reported hyperactivity and activity as measured by actigraphy in children with ADHD (Burley et al., 2022).

1.1.8 Body Mass Index.

Raw Body Mass Index (BMI) was calculated using measured height and weights using the standard formula:

$$\frac{703*W}{H^2}$$

Where W is the participants weight in lbs (averaged over up to 3 measurements) and H is the participant's height in inches. The raw value was then standardized, with adjustment for participant age and sex, using WHO norms as implemented by the R package zscorer (Myatt & Guevarra, 2019).

2 Code Supplement

This section includes R code used to generate all tables, figures, and statistics used in the main manuscript. Code is visible in the original Rmd document available (together with additional pre-processing code) on osf: https://osf.io/57xer/. The headings are hidden from the rendered document for legibility.

3 Supplementary Results

3.1 F-tests for discrete variable-Motion associations

In the manuscript, F tests associated with the discrete variables tested are omitted for brevity. These are printed in Tables 1-2.

3.2 Relation of participant characteristics to scan time (post scrubbing)

Participants have a variable number of low-motion frames available for analysis, contingent on the chosen motion threshold and participant motion. This variability may lead to heterogeneity in the stability of functional connectivity estimates between participants. Although five minutes of 'good' scan time is conventionally considered sufficient for a stable estimate, the following analysis examines the relation of scan length (post-scrubbing) to participant characteristics. The results are present in supplemental tables i and ii, which are available in a spreadsheet in the source OSF repository (https://osf.io/57xer/).

3.3 Multiply at Risk Cell Counts

Count of participants with multiple risk factors who were excluded (by condition) appear in Table 3-4.

3.4 Categorical Variable Table by Condition

See Table 5.

3.5 Missingness in Behavioral Data

Table 6 shows missingness counts in non-imaging variables with more than 50 missing cases.

3.6 Adjusted Models – Forest Plot

Figure 1-2 depict effects from the adjusted/condition models.

3.7 Sensitivity to Site/Scanner

Percent Excluded by site is shown in table 7.

Table 1: F tests for relation of discrete DVs with FD after scrubbing, by threshold (pt. 1)

Threshold	DV	F	DF_1	DF_2	MODEL_P
0.1mm	sex	40.6	1	9.37e+03	1.99e-10
0.1mm	household.income	20	5	8.6e+03	6.39e-20
0.1mm	p.edu	17.9	4	9.36e+03	1.3e-14
0.1mm	race_ethnicity.factor	27.5	4	9.36e+03	9.06e-23
0.1mm	ksads_factor	4.61	2	9.37e+03	0.00996
0.1mm	pds_category	2.36	4	9.29e+03	0.0512
0.2mm	sex	88	1	9.58e+03	7.92e-21
0.2mm	household.income	27.5	5	8.79e+03	9.8e-28
0.2mm	p.edu	27.7	4	9.56e+03	6.43e-23
0.2mm	race_ethnicity.factor	32.9	4	9.57e+03	2.95e-27
0.2mm	ksads_factor	6.75	2	9.57e+03	0.00118
0.2mm	pds_category	1.42	4	9.49e+03	0.224
0.3mm	sex	116	1	9.59e+03	7.92e-27
0.3mm	household.income	29.5	5	8.8e+03	8.34e-30
0.3mm	p.edu	31.7	4	9.57e+03	2.97e-26
0.3mm	race_ethnicity.factor	31.5	4	9.58e+03	4.18e-26
0.3mm	ksads_factor	6.94	2	9.59e+03	0.000977
0.3mm	pds_category	0.843	4	9.5e+03	0.498

Table 2: F tests for relation of discrete DVs with FD after scrubbing, by threshold (pt. 2)

Threshold	DV	F	DF_1	DF_2	MODEL_P
0.4mm	sex	128	1	9.59e+03	2.21e-29
0.4mm	household.income	29.5	5	8.8e+03	8.32e-30
0.4mm	p.edu	32.7	4	9.58e+03	3.97e-27
0.4mm	race_ethnicity.factor	28.8	4	9.58e+03	8.03e-24
0.4mm	ksads_factor	7.06	2	9.59e+03	0.000859
0.4mm	pds_category	1.15	4	9.5e+03	0.331
0.5mm	sex	136	1	9.59e+03	3.02e-31
0.5mm	household.income	28.8	5	8.8e+03	4.6e-29
0.5mm	p.edu	33.5	4	9.58e+03	7.92e-28
0.5mm	race_ethnicity.factor	26.5	4	9.59e+03	6.25e-22
0.5mm	ksads_factor	7	2	9.59e+03	0.000914
0.5mm	pds_category	1.12	4	9.51e+03	0.347

Table 3: Non-white (census) with psychopathology at z >= 1.5

F	T	C	.5	.4	.3	R	.2	.1
552	510	407	388	385	373	399	339	180

Table 4: Male participants with NIH toolbox total scores of z <= -1.5

F	T	C	.5	.4	.3	R	.2	.1
382	343	275	254	248	235	232	207	94

Table 5: Categorical Values at Each Level of Stringency (Subjects excluded with < 375 Frames), Pt 1

		Full		QC1		ABCC		0.5		0.4		0.3mm		QC2		0.2mm		0.1mm	
		n	%	n	%	n	%	n	%	n	%	n	%	n	%	n	%	n	%
Total	Total	11,876	1e+02	11,355	96	9,600	81	9,320	78	9,262	78	9,098	77	9,627	81	8,507	72	5.25e+03	44.2
Sex	F	5,680	48	5,444	48	4,575	48	4,475	48	4,458	48	4,408	48	4,792	50	4,199	49	2.7e+03	51.5
	M	6,196	52	5,911	52	5,025	52	4,845	52	4,804	52	4,690	52	4,835	50	4,308	51	2.55e+03	48.5
	Missing	0	0	0	0	0	0	0	0	0	0	0	0	0	0	0	0	0	0
Income	$100k to $200k	3,314	28	3,192	28	2,735	28	2,673	29	2,660	29	2,623	29	2,785	29	2,470	29	1.62e+03	30.9
	$0 to $25k	1,635	14	1,526	13	1,206	13	1,139	12	1,126	12	1,106	12	1,188	12	988	12	538	10.2
	$25k to $50k	1,588	13	1,524	13	1,291	13	1,250	13	1,239	13	1,212	13	1,277	13	1,135	13	676	12.9
	$50k to $75k	1,499	13	1,441	13	1,254	13	1,215	13	1,208	13	1,189	13	1,204	13	1,106	13	630	12
	$75k to $100k	1,572	13	1,508	13	1,316	14	1,281	14	1,276	14	1,250	14	1,315	14	1,188	14	773	14.7
	Over $200k	1,250	11	1,206	11	1,014	11	995	11	992	11	978	11	1,056	11	930	11	626	11.9
	Missing	1,018	8.6	958	8.4	784	8.2	767	8.2	761	8.2	740	8.1	802	8.3	690	8.1	385	7.33
Parent Ed.	College Degree	3,015	25	2,877	25	2,504	26	2,441	26	2,429	26	2,393	26	2,486	26	2,268	27	1.44e+03	27.4
	< HS	593	5	551	4.9	388	4	370	4	365	3.9	359	3.9	439	4.6	324	3.8	173	3.3
	HS Graduate	1,132	9.5	1,068	9.4	855	8.9	815	8.7	809	8.7	786	8.6	846	8.8	721	8.5	373	7.1
	Some College	3,079	26	2,957	26	2,528	26	2,432	26	2,410	26	2,361	26	2,451	25	2,179	26	1.29e+03	24.6
	Graduate Degree	4,043	34	3,889	34	3,313	35	3,250	35	3,238	35	3,188	35	3,396	35	3,007	35	1.97e+03	37.5
	Missing	14	0.12	13	0.11	12	0.12	12	0.13	11	0.12	11	0.12	9	0.093	8	0.094	3	0.0571
Race/Ethnicity	White	6,180	52	5,945	52	5,238	55	5,104	55	5,075	55	4,999	55	5,146	53	4,710	55	3.07e+03	58.5
	Black	1,784	15	1,672	15	1,338	14	1,279	14	1,269	14	1,241	14	1,313	14	1,122	13	569	10.8
	Hispanic	2,411	20	2,312	20	1,845	19	1,799	19	1,788	19	1,746	19	1,966	20	1,630	19	960	18.3
	Asian	252	2.1	236	2.1	180	1.9	175	1.9	175	1.9	168	1.8	196	2	155	1.8	101	1.92
	Other	1,247	11	1,188	10	997	10	961	10	953	10	942	10	1,004	10	888	10	545	10.4
	Missing	2	0.017	2	0.018	2	0.021	2	0.021	2	0.022	2	0.022	2	0.021	2	0.024	2	0.0381
Trauma Count	0	7,723	65	7,383	65	6,275	65	6,102	65	6,059	65	5,957	65	6,295	65	5,586	66	3.47e+03	66.1
	1	3,004	25	2,882	25	2,422	25	2,345	25	2,330	25	2,285	25	2,437	25	2,147	25	1.34e+03	25.5
	>2	1,149	9.7	1,090	9.6	903	9.4	873	9.4	873	9.4	856	9.4	895	9.3	774	9.1	440	8.38
	Missing	0	0	0	0	0	0	0	0	0	0	0	0	0	0	0	0	0	0
Puberty	pre-pubertal	5,938	50	5,690	50	4,845	50	4,707	51	4,671	50	4,575	50	4,818	50	4,284	50	2.67e+03	50.9
	early puberty	2,815	24	2,680	24	2,275	24	2,207	24	2,195	24	2,160	24	2,268	24	1,998	23	1.26e+03	24
	mid puberty	2,798	24	2,681	24	2,253	23	2,190	23	2,181	24	2,150	24	2,289	24	2,025	24	1.2e+03	22.8
	late puberty	188	1.6	180	1.6	135	1.4	127	1.4	126	1.4	126	1.4	152	1.6	118	1.4	75	1.43
	post pubertal	12	0.1	10	0.088	10	0.1	9	0.097	9	0.097	9	0.099	7	0.073	7	0.082	3	0.0571
	Missing	125	1.1	114	1	82	0.85	80	0.86	80	0.86	78	0.86	93	0.97	75	0.88	43	0.819

Table 6: Behavioral and Demographic variables with >50 missing values

Variable	n Missing	% missing
Child Opportunity Index	1093	9.2
Household Income	1018	8.57
Area Disadvantage Index	863	7.27
NIH Toolbox Total	397	3.34
NIH Toolbox Crystalized	338	2.85
WISC V	249	2.1
ethn.iden.hisp	153	1.29
NIH Toolbox Flanker	153	1.29
Pubertal Status	125	1.05

Table 7: Sites with Highest % Excluded, by Condition

Site	Condition	% Excluded
Pittsburgh, Pa	DAIC inclusion	32.5
Pittsburgh, Pa	Current FastQC	30.3
Los Angeles, CA (CHLA)	ABCC <.2mm FD	75.6
Baltimore, MD	ABCC	72.7

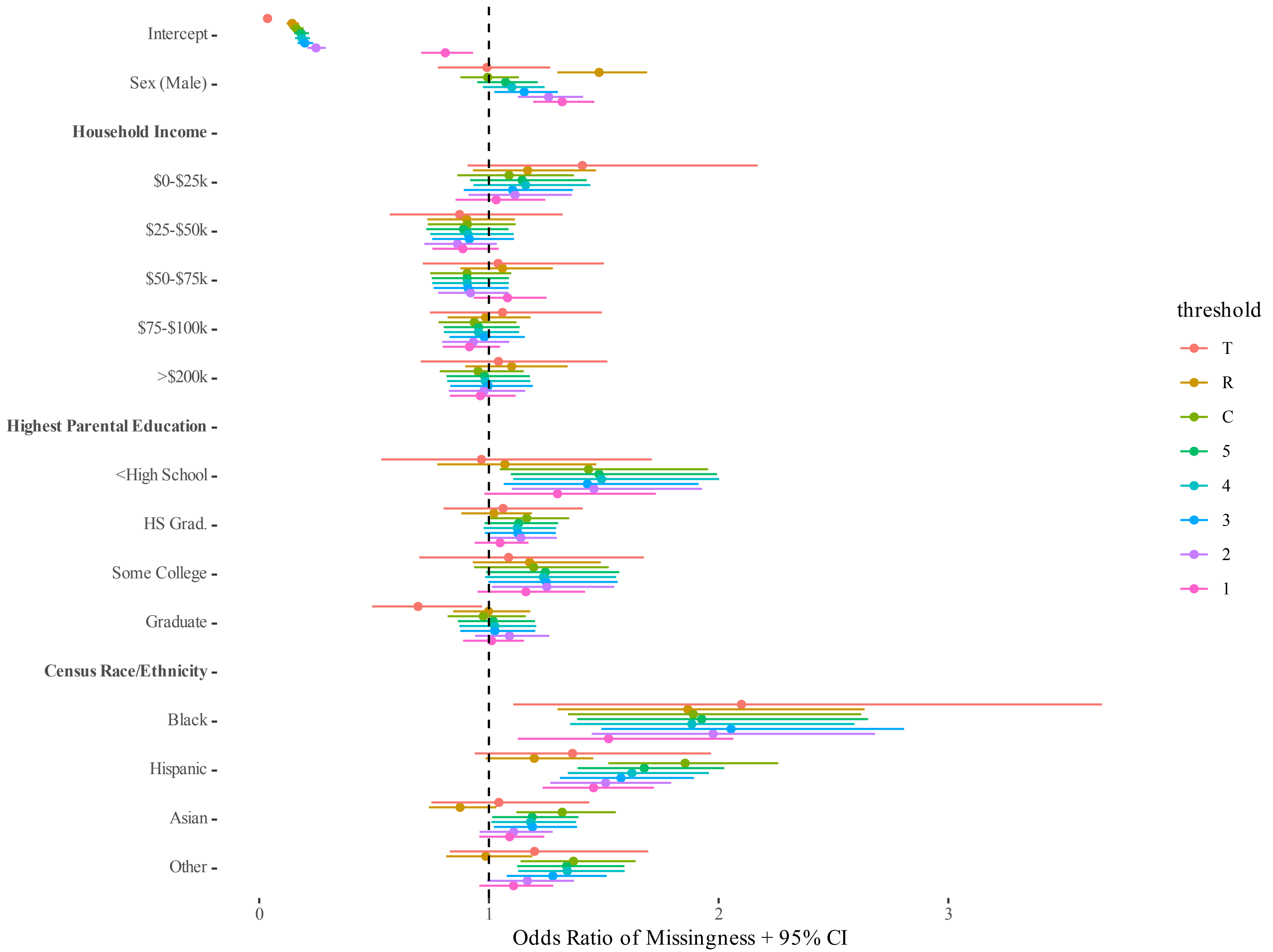

Figure 1: Odds ratios for missingness by threshold – Adjusted Models (part 1)

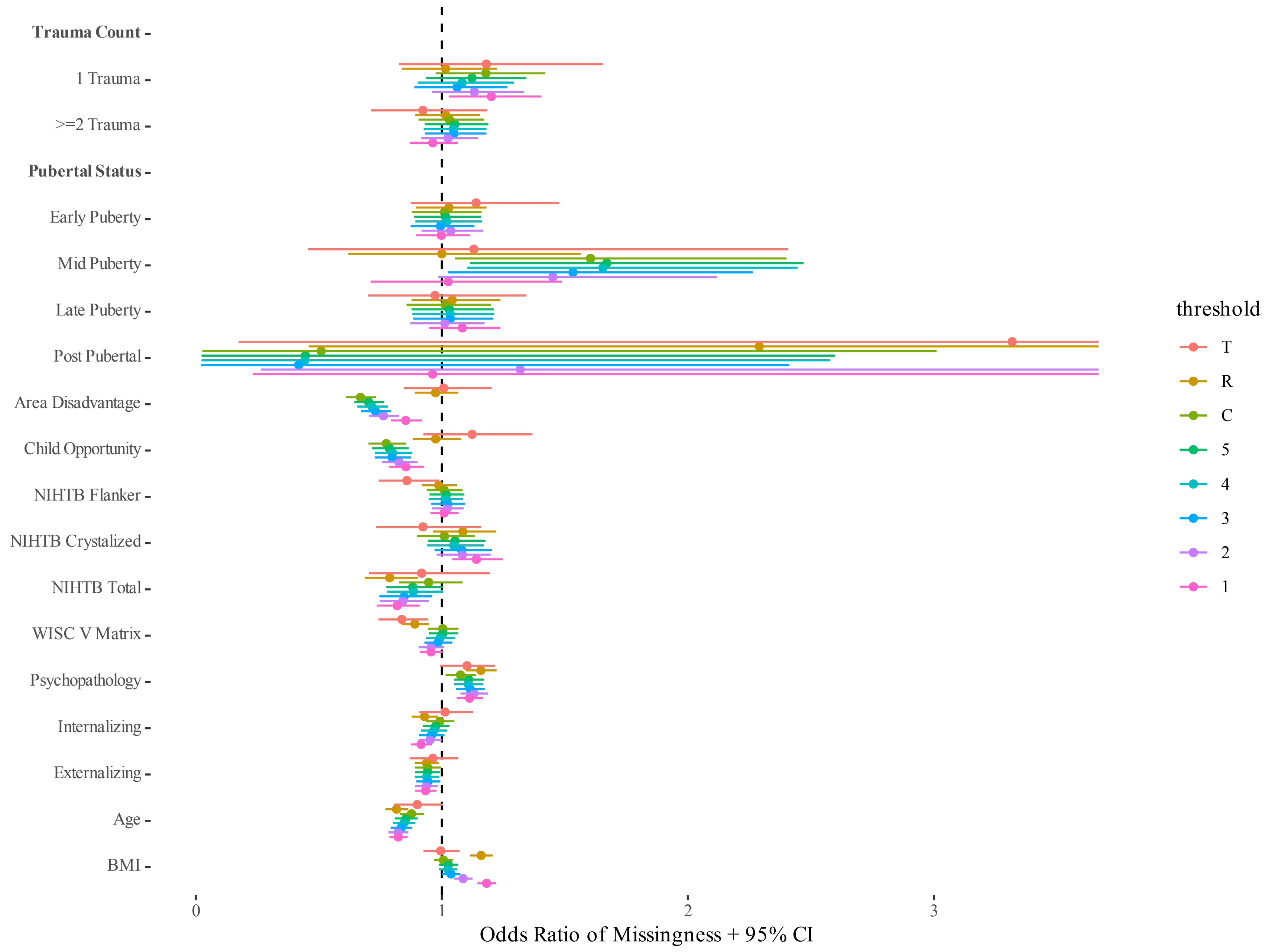

Figure 2: Odds ratios for missingness by threshold – Adjusted Models (part 2)

3.7.1 Sensitivity to Site/Scanner Table

Table 8 shows fixed effects of a 'scanner type' variable in 3 of our 'adjusted' models both with and without control for site.

Table 8: OR of scanner type effect, with and without site control, in condition adjusted models [95% CI]. ***: p<.001

	Without Site Control		
Scanner (ref: Philips Achieva)	ABCC	QC2	0.2mm
GE Discovery MR750	.86 [.72–1.04]	.56*** [.46–.69]	.70*** [.59–.84]
Philips Ingenia	.20*** [.13–.31]	.87 [.63–1.19]	.42*** [.30–.57]
Siemens Prisma	.15*** [.12–.20]	.27*** [.22–.33]	.20*** [.17–.25]
Siemens Prisma fit	.39*** [.32–.47]	.43*** [.36–.53]	.43*** [.36–.51]
	With Site Control		
GE Discovery MR750	3894.69 [1.69e-14–NA]	44437.50 [.00–NA]	13726.89 [.00–NA]
Philips Ingenia	8698.06 [4.91e-09–NA]	7.24e+09 [5.44e-11–NA]	9.30e+09 [.01–NA]
Siemens Prisma	3109.47 [1.16e-14–NA]	8613.49 [.00–NA]	15297.36 [.00–NA]
Siemens Prisma fit	6030.51 [2.24e-14–NA]	8257.41 [.00–NA]	16617.52 [.00–NA]

3.7.2 Sensitivity to Site/Scanner Forest Plots

Figures 3-4 show forest plots of ORs with a significant (p<.05) model term in either the site-controlled or the site un-controlled adjusted model. They are here to visualize the effects of site control on the adjusted models.

3.8 Current Fast Track QC vs DAIRC recommendations

We state in the text that the currently published fastqc is non-overlapping with DAIRC inclusion recommendations. Tables 9 and 10 illustrate that non-overlap.

The variable 'fastqcok' is TRUE for participants who have at least one T1, rs-fMRI, and field map image marked useable in the current (Last modified 09/09/2019) abcd_fastqc01.csv file. These recommendations are non overlapping with the tabular data list as well as DAIRC recommendations:

3.9 Modeling Trends Across Conditions (H2)

This model specifically looks at changes associated with motion threshold choice in the ABCC data. In the pre-registration, we originally proposed a mixed effects logistic model where each participant had a case for each motion threshold, and the percentage of data missing from each threshold was used as a within-subject variable. In simulation, we discovered a number of problems with this approach:

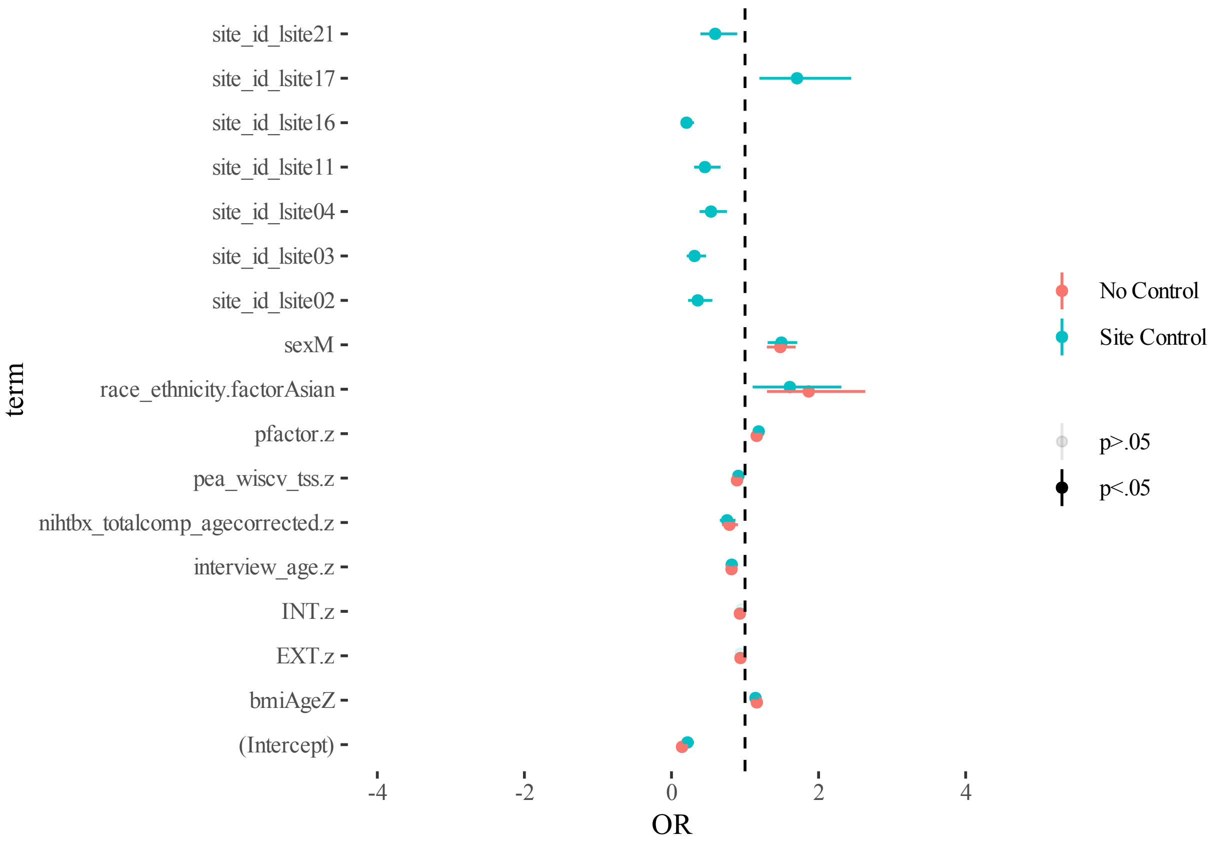

Figure 3: Significant ORs predicting DAIRC Recommended missingness with and without site control

Table 9: FastQC vs Tabulated Data Inclusion/Exclusion

	Excluded (Tabulated)	Included (Tabulated)
Excluded (FastQC)	410	1378
Included (FastQC)	111	9977

Table 10: FastQC vs DAIRC recommended Inclusion/Exclusion

	Excluded (DAIRC Recommended)	Included (DAIRC Recommended)
Excluded (FastQC)	822	966
Included (FastQC)	1427	8661

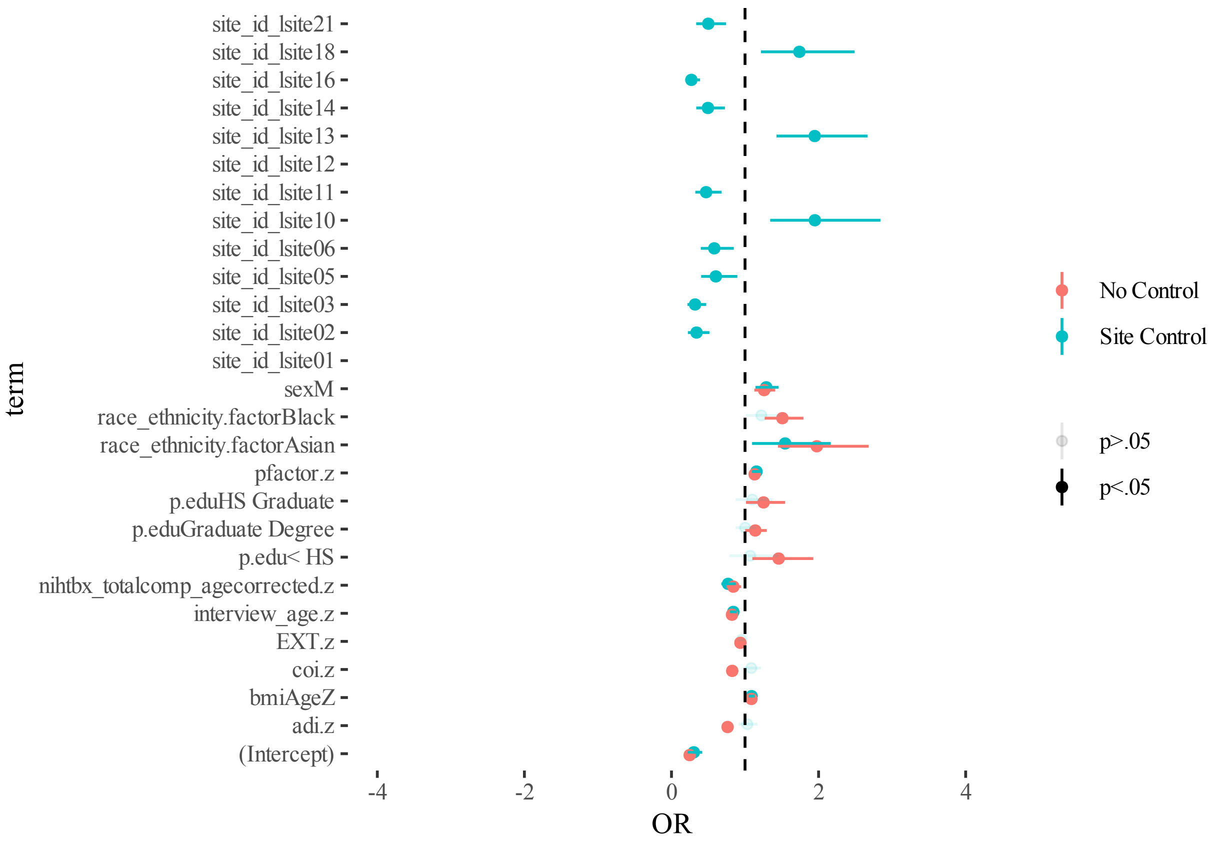

Figure 4: Significant ORs predicting .2mm missingness with and without site control

1. The proposed mixed effects logistic regression created very poor predictions and model fits in simulation, and often did not converge. A linear model of probability proved more stable and accurate and we determined to use that approach (a logistic model is also presented below, but it is similarly flawed).
2. We proposed that an interaction between a variable and the 'pmiss' variable would indicate that bias was worsening as more data was excluded. However, in simulating this approach we discovered that in the case that there was a bias but it did not worsen as more data was excluded, as, for example, if 2 males were excluded for every female, this would surface as a linear interaction between pmiss and sex in the model. Instead, a worsening bias would be indicated by an interaction between a variable and a polynomial of pmiss, which would indicate a curvilinear trajectory.

(notes from the simulation are available in the osf repository)

An important remaining question from the simulation study was whether to include an intercept and main effects in the model. As, at pmiss=0, the probability of exclusion ought to be 0 for everyone, one could argue the intercept should be omitted. Model fit is compared between the two models in Table 11. The 'full' model has superior fit characteristics across indices. The GLM model, which is closer to what we originally proposed, does not converge and is not presented.

Table 11: Comparison of Model Performance Indices

Name	Model	AIC (weights)	AICc (weights)	BIC (weights)	R2 (cond.)	R2 (marg.)	ICC	RMSE	Sigma
m.h2	lmerModLmerTest	-1.8e+05 (>.999)	-1.8e+05 (>.999)	-1.8e+05 (>.999)	0.91	0.19	0.89	0.18	0.19
m.h2.stripped	lmerModLmerTest	3.5e+05 (<.001)	3.5e+05 (<.001)	3.5e+05 (<.001)	0.51	0.28	0.31	0.34	0.34

```
##  [1] "Linear mixed model fit by REML. t-tests use Satterthwaite's method ["
##  [2] "lmerModLmerTest]"
##  [3] "Formula: "
##  [4] "missing ~ poly(pmiss, 2) + sex + sex:poly(pmiss, 2) + household.income +  "
##  [5] "    household.income:poly(pmiss, 2) + p.edu + p.edu:poly(pmiss,  "
##  [6] "    2) + race_ethnicity.factor + race_ethnicity.factor:poly(pmiss,  "
##  [7] "    2) + ksads_factor + ksads_factor:poly(pmiss, 2) + pds_category +  "
##  [8] "    pds_category:poly(pmiss, 2) + adi.z + adi.z:poly(pmiss, 2) +  "
##  [9] "    coi.z + coi.z:poly(pmiss, 2) + nihtbx_flanker_agecorrected.z +  "
## [10] "    nihtbx_flanker_agecorrected.z:poly(pmiss, 2) + nihtbx_cryst_agecorrected.z +  "
## [11] "    nihtbx_cryst_agecorrected.z:poly(pmiss, 2) + nihtbx_totalcomp_agecorrected.z +  "
## [12] "    nihtbx_totalcomp_agecorrected.z:poly(pmiss, 2) + pea_wiscv_tss.z +  "
## [13] "    pea_wiscv_tss.z:poly(pmiss, 2) + pfactor.z + pfactor.z:poly(pmiss,  "
## [14] "    2) + INT.z + INT.z:poly(pmiss, 2) + EXT.z + EXT.z:poly(pmiss,  "
## [15] "    2) + interview_age.z + interview_age.z:poly(pmiss, 2) + bmiAgeZ +  "
## [16] "    bmiAgeZ:poly(pmiss, 2) + (pmiss | subjectkey)"
## [17] "   Data: dflong"
## [18] ""
## [19] "REML criterion at convergence: -176817.8"
## [20] ""
## [21] "Scaled residuals: "
## [22] "    Min      1Q  Median      3Q     Max "
## [23] "-5.5260 -0.3032  0.0429  0.2260  5.0294 "
## [24] ""
## [25] "Random effects:"
```

```
## [26] " Groups     Name        Variance Std.Dev. Corr "
## [27] " subjectkey (Intercept) 0.26699  0.5167         "
## [28] "            pmiss       0.29719  0.5452   -0.94"
## [29] " Residual               0.03466  0.1862         "
## [30] "Number of obs: 463488, groups:  subjectkey, 9088"
## [31] ""
```

See Table 12-13 for the fixed effects from the omnibus model.

3.9.1 H2 Marginal Means plotting (Figure 5)

The poor fit of the omnibus model is shown in Figure 5, a plot with both model predicted marginal means and the average inclusion within each factor level and motion threshold (here, lines are the model marginal means and points are the averages from the data).

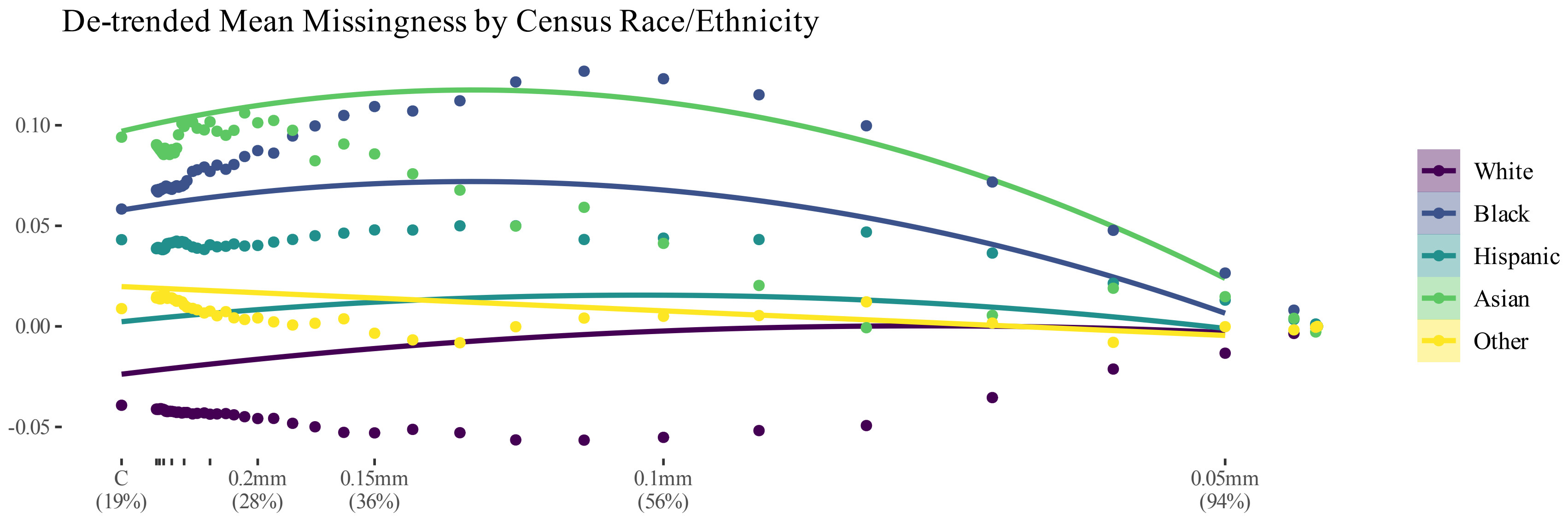

Figure 5: Marginal Means Plot of Race/Ethnicity on exclusion by condition (adjusted)

Figure 6 shows a similar plot but in a bivariate model – fit is much improved, indicating that distortions in the first plot are most likely due to interference from other variables. Regardless, H2 is generally not supported by these results. The descriptive data is probably more helpful.

Figures 7-9 reproduce Manuscript figures 5 for all of the other demographic and behavioral variables (in groups of 4):

3.10 Association of QC missingness with Propensity weighting.

Gard (2020) notes that analyses using propensity weighting can be biased when missing data is correlated with population weights. They presented data that association between missingness and population weights are small when considering structural and task-based brain data from ABCD. Here, we repeat these analyses considering rs-fMRI data in each condition.

Table 12: H2 / Omnibus model fixed effects, pt. 1

	B	CI.l	CI.u	*	B	CI.l	CI.u	*	B	CI.l	CI.u	*
	Intercept				pmiss				pmiss^2			
	1.36	1.33	1.39	***	1.40e+87	9.68e+83	2.04e+90	***	4.66e+08	1.18e+08	1.84e+09	***
	Main Eff.				Interaction				Q.Interaction			
Sex (Male)	1.03	1.01	1.05	***	1.73	.01	329.14		.00	6.56e-08	.00	***
$0-$25k	1.02	.99	1.05		.00	.00	72.33		.69	.11	4.32	
Household Income (ref: $100-$200k)												
$25-$50k	.98	.96	1.01		105.65	.02	507779.58		44.64	9.04	220.55	***
$50-$75k	.99	.97	1.02		1165.69	.56	2426422.31		.01	.00	.06	***
$75-$100k	.99	.97	1.01		1.98	.00	2541.64		25.95	6.74	99.95	***
>$200k	.99	.97	1.02		.64	.00	1587.92		12.81	2.94	55.89	***
Highest Parental Education (ref: College Degree)												
<High School	1.07	1.02	1.12	**	8.22e-09	6.06e-15	.01	**	.00	.00	.05	***
HS Grad.	1.04	1.00	1.07	*	.00	2.26e-08	18.33		.00	.00	.01	***
Some College	1.00	.98	1.03		1.64	.00	1525.01		.06	.02	.22	***
Graduate	1.02	1.00	1.04	*	.08	.00	29.44		.05	.02	.15	***
Census Race/Ethnicity (ref: White)												
Black	1.07	1.05	1.10	***	3.79e-09	7.89e-13	.00	***	.00	.00	.01	***
Hispanic	1.02	1.00	1.04	*	.00	.00	1.51		.41	.11	1.46	
Asian	1.12	1.06	1.17	***	8.26e-12	.00	.00	**	.00	.00	.00	***
Other	1.03	1.01	1.05	*	.00	5.80e-09	.02	**	18.54	4.43	77.57	***
KSADS Trauma Count (ref: 0 Exposures)												
1 Trauma	1.00	.99	1.02		.23	.00	43.30		5.69	2.12	15.29	***
>=2 Trauma	1.02	.99	1.04		.07	.00	205.22		.00	.00	.00	***

Table 13: H2 / Omnibus model fixed effects, pt. 2

	B	CI.l	CI.u	*	B	CI.l	CI.u	*	B	CI.l	CI.u	*
	Intercept				pmiss				pmiss^2			
	1.36	1.33	1.39	***	1.40e+87	9.68e+83	2.04e+90	***	4.66e+08	1.18e+08	1.84e+09	***
	Main Eff.				Interaction				Q.Interaction			
Pubertal Status (ref: pre-pubertal)												
Early Puberty	1.00	.99	1.02		1.19	.00	359.61		.28	.10	.83	*
Mid Puberty	1.01	.99	1.03		2.05	.00	2067.75		.03	.01	.10	***
Late Puberty	1.06	1.00	1.13	*	1.91e-12	.00	.00	**	3926.89	109.53	140784.45	***
Post Pubertal	.94	.75	1.19		5.18e+15	.00	2.07e+48		1.14e-13	.00	.00	***
Area Disadvantage	.95	.94	.97	***	597703.26	13593.00	2.63e+07	***	2.14	1.05	4.36	*
Child Opportunity	.97	.95	.98	***	4424.30	64.41	303924.17	***	11.79	5.31	26.16	***
NIHTB Flanker	1.00	.99	1.01		1.25	.07	23.95		.68	.39	1.19	
NIHTB Crystalized	1.01	1.00	1.03	*	7.21	.07	784.75		.00	.00	.00	***
NIHTB Total	.97	.96	.99	**	1.21	.00	301.16		32303.00	11419.24	91379.42	***
WISC V Matrix	1.00	.99	1.00		.68	.05	8.64		20.63	12.78	33.30	***
Psychopathology	1.02	1.01	1.03	***	.04	.00	.41	**	.01	.01	.02	***
Internalizing	.99	.98	1.00	*	.85	.08	9.31		104.30	66.47	163.66	***
Externalizing	.99	.98	1.00	**	5.66	.53	59.92		6.72	4.31	10.49	***
Age	.97	.96	.98	***	23.98	2.39	240.40	**	3247.86	2103.56	5014.63	***
BMI	1.01	1.01	1.02	***	18.73	3.73	94.21	***	.00	.00	.00	***

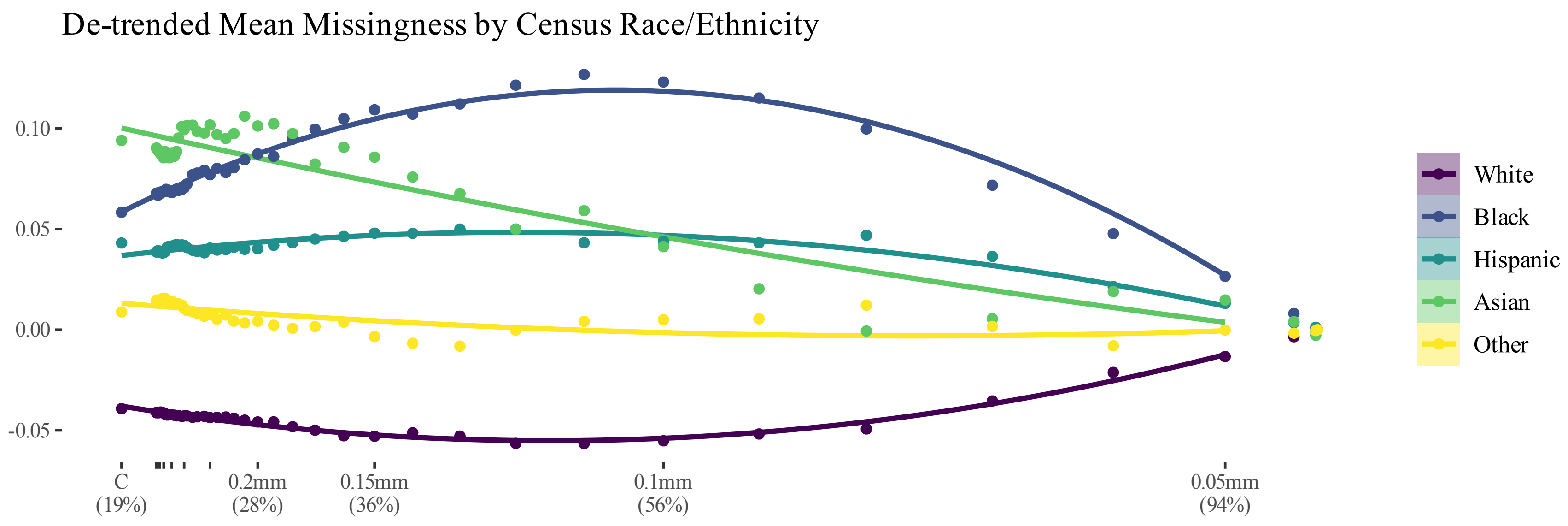

Figure 6: Marginal Means Plot of Race/Ethnicity on exclusion by condition (bivariate)

To the best of our knowledge, Gard et al. used Welch's two-sample t-tests to examine differences in propensity weight between included and excluded groups, and we will do the same here. Results from these tests are shown in table 14.

3.11 Association of Exclusion with Expanded Demographic Variables

Available information on household income and (especially) race/ethnicity was greatly simplified in the main analysis for the purpose of brevity and to facilitate comparison with existing literature. For the sake of completeness and inclusivity, we present more nuanced codings of these variables here.

3.11.1 Household Income

Table 15 and 16 presents descriptives and a bivariate model for a SES variable inclusive of all income bins available in the ABCD dataset.

3.11.2 Detailed Race/Ethnicity

Census Race/Ethnicity Categories were used in the manuscript for the sake of brevity and because they are conventional and easy to compare to other work (which is of primary importance in a work commenting on methods). However, we acknowledge that the census categories are reductive both in the number of identifications present and in the lack of nuance they present (i.e., inability to pick multiple categories). ABCD collects information on participant race/ethnicity in considerably more detail, specifically as a series of yes/no questions about particular identities. We here present a comparison tables between the two measures, descriptives of the more granular race/ethnicity data, and bivariate models using those variables.

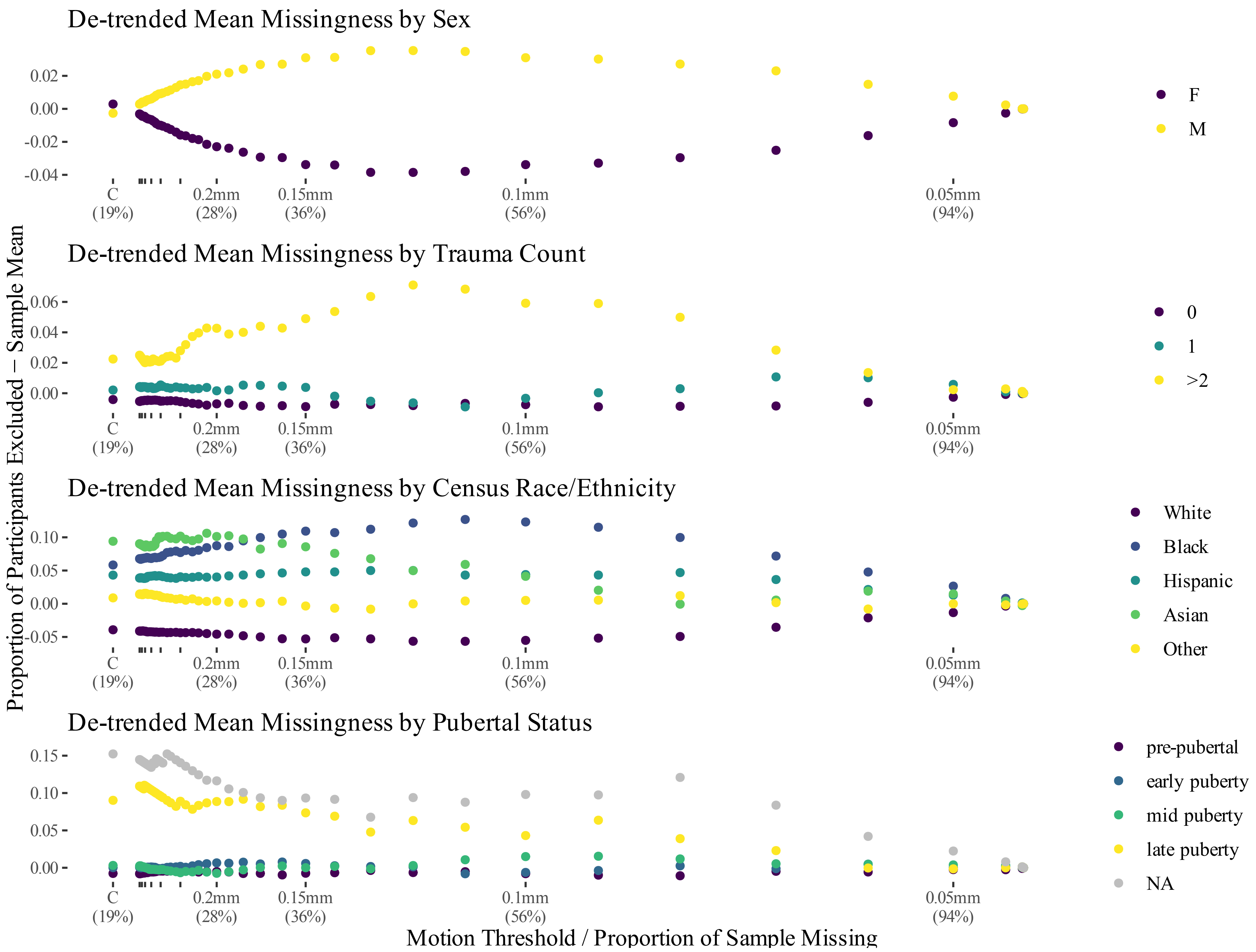

Figure 7: Mean Inclusion/Exclusion by Supplemental Variables and condition (part 1)

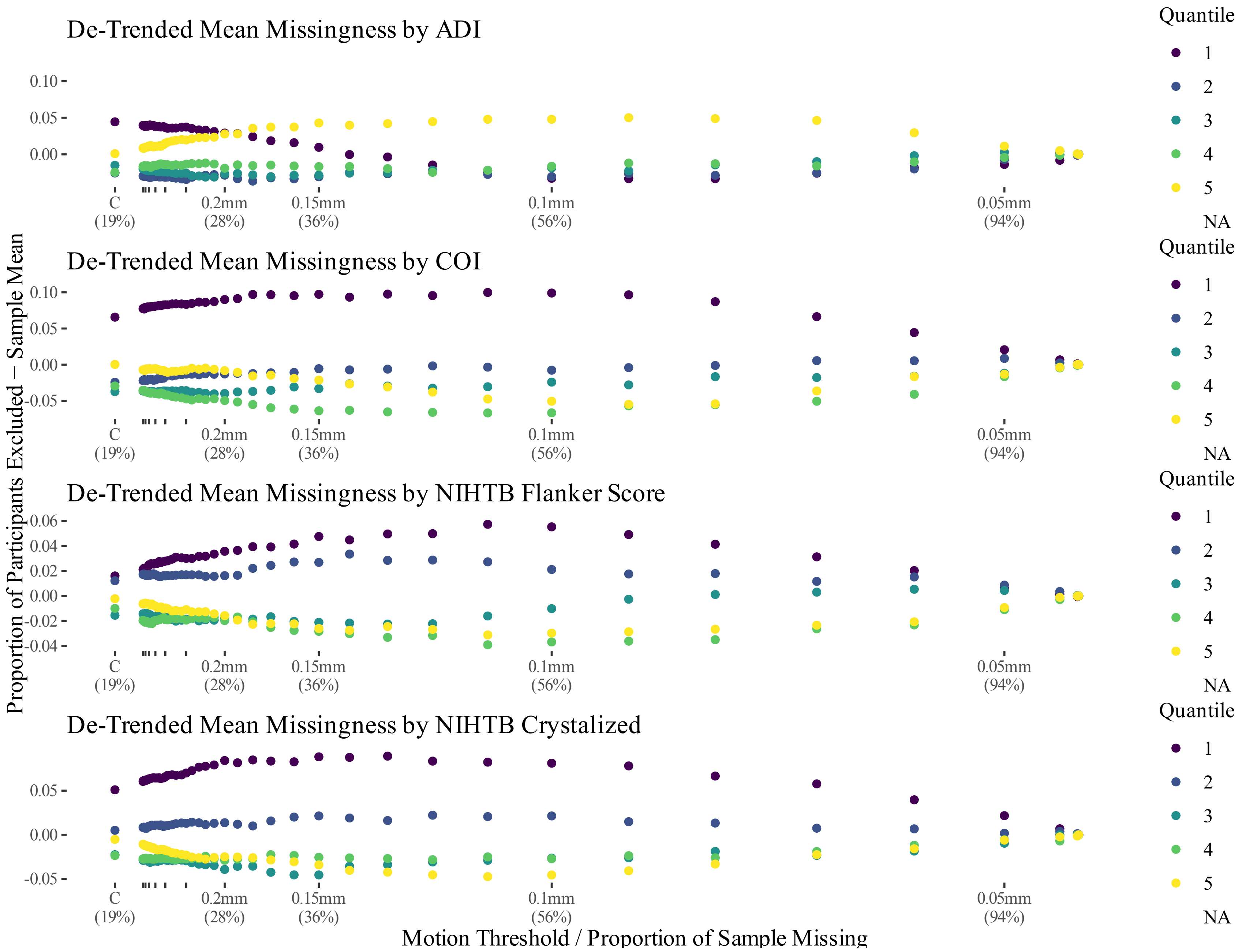

Figure 8: Mean Inclusion/Exclusion by Supplemental Variables and Condition (part 2)

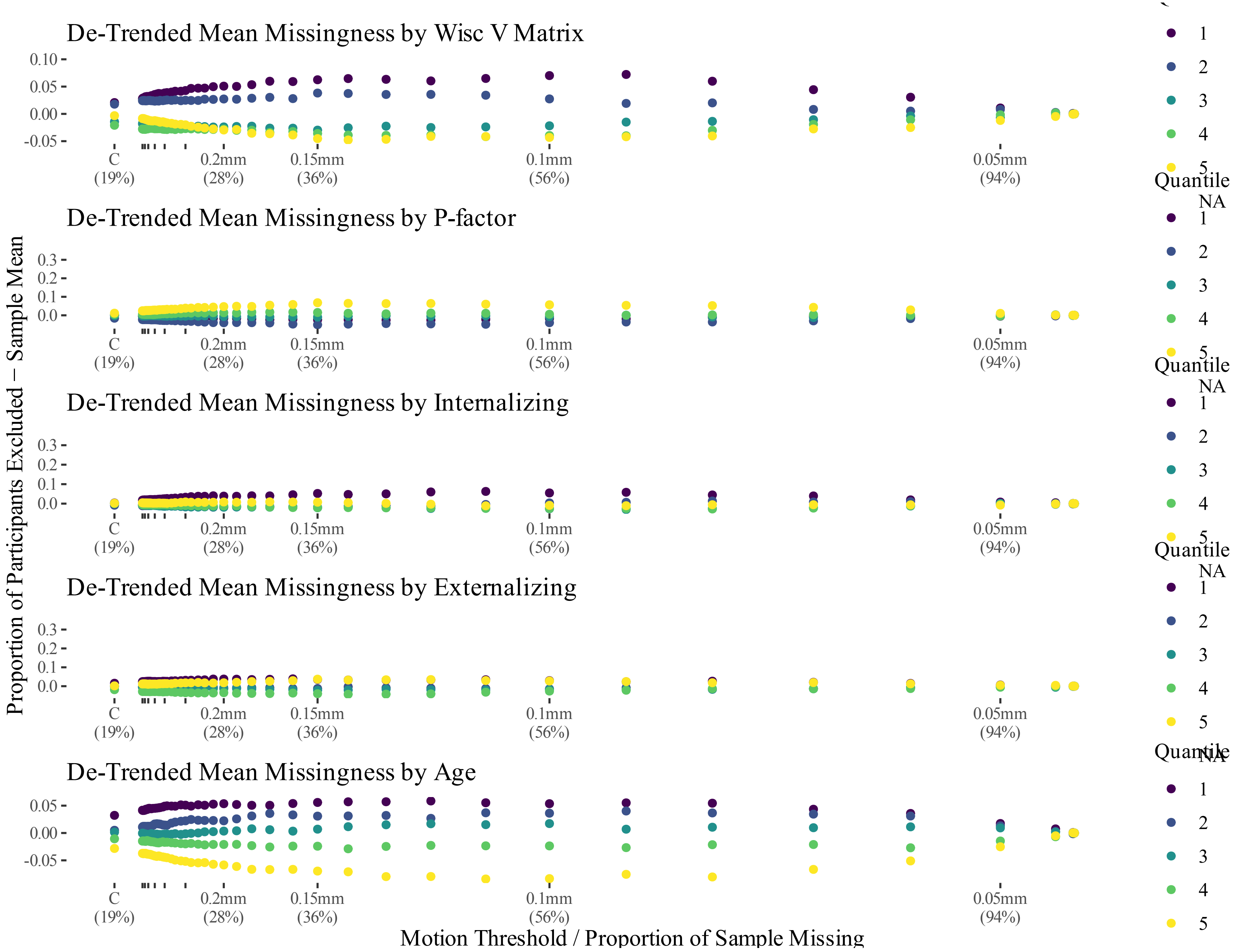

Figure 9: Mean Inclusion/Exclusion by Supplemental Variables and Condition (part 3)

Table 14: Propensity weights of included vs. excluded participants by condition; t-test parameters

	delta	Mexcluded	Mincluded	t	p	df	ci.low	ci.high	d
ABCD 4 Tabulated (T)	-0.487	691	691	-0.0309	0.975	568	-31.5	30.5	-0.00139
ABCC (C)	-3.68	688	692	-0.472	0.637	3.64e+03	-19	11.6	-0.0105
ABCC < 0.5mm	-4.63	688	692	-0.617	0.537	4.33e+03	-19.3	10.1	-0.0132
ABCC < 0.4mm	-3.49	689	692	-0.467	0.64	4.46e+03	-18.1	11.2	-0.00994
ABCC < 0.3mm	-2.93	689	692	-0.4	0.689	4.89e+03	-17.3	11.4	-0.00835
ABCD 4 Recommended (R)	22.4	709	687	2.72	0.00652	3.38e+03	6.25	38.5	0.0637
ABCD < 0.2mm	8.1	697	689	1.16	0.248	6.44e+03	-5.63	21.8	0.0231
ABCD < 0.1mm	41.6	710	668	6.42	1.45e-10	1.12e+04	28.9	54.3	0.119

Table 15: Granular Household Income at Each Level of Stringency (Subjects excluded with < 375 Frames)

	Full		QC1		ABCC		0.5		0.4		0.3mm		QC2		0.2mm		0.1mm	
	n	%	n	%	n	%	n	%	n	%	n	%	n	%	n	%	n	%
Total	11,876	1e+02	11,355	96	9,600	81	9,320	78	9,262	78	9,098	77	9,627	81	8,507	72	5.25e+03	44.2
Less than $5k	417	3.5	379	3.3	289	3	279	3	273	2.9	266	2.9	280	2.9	228	2.7	125	2.38
$5k through $11.9k	421	3.5	386	3.4	311	3.2	287	3.1	286	3.1	283	3.1	293	3	252	3	130	2.48
$12k through $15.9k	273	2.3	266	2.3	213	2.2	200	2.1	199	2.1	196	2.2	220	2.3	184	2.2	102	1.94
$16k through $24.9k	524	4.4	495	4.4	393	4.1	373	4	368	4	361	4	395	4.1	324	3.8	181	3.45
$25k through $34.9k	654	5.5	628	5.5	521	5.4	503	5.4	499	5.4	491	5.4	537	5.6	459	5.4	269	5.12
$35k through $49.9k	934	7.9	896	7.9	770	8	747	8	740	8	721	7.9	740	7.7	676	7.9	407	7.75
$50k through $74.9k	1,499	13	1,441	13	1,254	13	1,215	13	1,208	13	1,189	13	1,204	13	1,106	13	630	12
$75k through $99.9k	1,572	13	1,508	13	1,316	14	1,281	14	1,276	14	1,250	14	1,315	14	1,188	14	773	14.7
$100k through $200k	3,314	28	3,192	28	2,735	28	2,673	29	2,660	29	2,623	29	2,785	29	2,470	29	1.62e+03	30.9
$200k and greater	1,250	11	1,206	11	1,014	11	995	11	992	11	978	11	1,056	11	930	11	626	11.9
Missing	1,018	8.6	958	8.4	784	8.2	767	8.2	761	8.2	740	8.1	802	8.3	690	8.1	385	7.33

Table 16: Bivariate Model of Granular Household Income and Missingness by Condition. Ref: $100k-$199k

	T	C	0.5	0.4	0.3	R	0.2	0.1
Household Income (ref: $100-$200k)	OR [95% CI]	OR [95% CI]	OR [95% CI]	OR [95% CI]	OR [95% CI]	OR [95% CI]	OR [95% CI]	OR [95% CI]
$5k through $11.9k	.90 [.56-1.46]	.80 [.59-1.08]	.94 [.71-1.26]	.89 [.67-1.19]	.86 [.65-1.14]	.89 [.67-1.19]	.81 [.61-1.06]	.96 [.71-1.29]
$12k through $15.9k	.26** [.11-.56]	.64* [.44-.90]	.74 [.53-1.03]	.70* [.50-.98]	.69* [.50-.96]	.49*** [.34-.70]	.58*** [.42-.80]	.72* [.52-.99]
$16k through $24.9k	.58* [.35-.96]	.75 [.56-1.00]	.82 [.62-1.08]	.80 [.61-1.06]	.80 [.61-1.04]	.67** [.50-.89]	.74* [.57-.97]	.81 [.61-1.07]
$25k through $34.9k	.41*** [.24-.69]	.58*** [.43-.76]	.61*** [.46-.80]	.59*** [.45-.77]	.58*** [.45-.76]	.45*** [.33-.59]	.51*** [.40-.66]	.61*** [.47-.79]
$35k through $49.9k	.42*** [.27-.67]	.48*** [.37-.63]	.51*** [.39-.66]	.50*** [.38-.64]	.52*** [.40-.67]	.54*** [.41-.69]	.46*** [.36-.59]	.55*** [.43-.71]
$50k through $74.9k	.40*** [.26-.62]	.44*** [.34-.57]	.47*** [.37-.60]	.46*** [.36-.58]	.46*** [.36-.58]	.50*** [.39-.64]	.43*** [.34-.54]	.59*** [.47-.74]
$75k through $99.9k	.42*** [.28-.65]	.44*** [.34-.56]	.46*** [.36-.58]	.44*** [.35-.56]	.45*** [.36-.57]	.40*** [.31-.51]	.39*** [.31-.49]	.44*** [.35-.56]
$100k through $200k	.38*** [.26-.56]	.48*** [.38-.60]	.48*** [.39-.61]	.47*** [.37-.58]	.46*** [.37-.58]	.39*** [.31-.49]	.41*** [.33-.51]	.45*** [.36-.56]
$200k and greater	.36*** [.23-.57]	.53*** [.41-.68]	.52*** [.41-.66]	.49*** [.39-.63]	.49*** [.39-.62]	.38*** [.29-.49]	.42*** [.33-.52]	.43*** [.34-.54]

3.11.2.1 Descriptives

Table 17 describes the many race/ethnicity endorsement variables available in ABCD, cross-referenced against the census race/ethnicity variable used in the manuscript.

Table 18 show descriptives of these variables by condition.

3.11.2.2 Bivariate Models

Finally, Tables 19-21 depict bivariate models for each race/ethnicity endorsement variable by condition.

4 Changes since pre-registration

The following changes were made to the analysis plan post-registration:

- BMI was added as a study variable, to enhance comparibility with Cosgrove et. al. (2020) and reflect the known relation between BMI and in-scanner motion.
- The Behavioral Inhibition scale was removed. It is not available in the ABCD baseline data and was included in error.
- More data was excluded prior to motion filtering than expected and there were more differences in inclusion criteria between ABCD versions than expected. Consequently, three 'QC' conditions were added in addition to the motion thresholds, to provide additional detail to inform study design.
- The originally planned approach to evaluate H2 was found to be infeasible (See 'H2 Model,' above). In practice, a visual inspection of the data did not support H2 (see H2 Marginal Means Plotting). Specifically, because so much data was missing, biases in the missing data were self-correcting as more data was excluded. I.e., in the event that males were more likely to be excluded than females (which appears to be true in this dataset), the bias is strongest as data is first excluded from the dataset. Once a significantly larger proportion of males have been excluded than females, the over-representation of females in the sample results in their being more likely to be excluded. At the extreme, when all of the data is excluded, there is no bias).

Here, we present the analyses originally proposed as written, excepting H2. Specifically, we present results from adjusted models without the BMI variable in the motion scrubbing conditions in Tables 22-23.

4.1 Save Tables

Supplemental tables are additionally available in an attached xlsx file.

5 Supplemental References

Achenbach, T. M., Dumenci, L., & Rescorla, L. A. (2002). Ten-year comparisons of problems and competencies for national samples of youth: Self, parent, and teacher reports. *Journal of Emotional and Behavioral Disorders, 10*(4), 194–203. doi:10.1177/10634266020100040101

Akshoomoff, N., Beaumont, J. L., Bauer, P. J., Dikmen, S. S., Gershon, R. C., Mungas, D., Slotkin, J., Tulsky, D., Weintraub, S., Zelazo, P. D., & Heaton, R. K. (2013). Nih Toolbox Cognition Battery (cb): Composite Scores of Crystallized, Fluid, and Overall Cognition. *Monographs of the Society for Research in Child Development, 78*(4), 119–132. doi:10.1111/mono.12038

Brislin, S. J., Martz, M. E., Joshi, S., Duval, E. R., Gard, A., Clark, D. A., Hyde, L. W., Hicks, B. M., Taxali, A., Angstadt, M., Rutherford, S., Heitzeg, M. M., & Sripada, C. (2021). Differentiated nomological networks of internalizing, externalizing, and the general factor of psychopathology ('p factor') in emerging adolescence in the ABCD study. *Psychological Medicine*, 1–11. doi:10.1017/S0033291720005103

Table 17: Percentage Endorsing Granular Race/Ethnicity Variables within Census Race/Ethnicity Groups

	Census Race/Ethnicity				
	White	Black	Hispanic	Asian	Other
White	99.9%	0.0%	68.0%	0.0%	79.3%
Black	0.0%	99.8%	8.9%	0.0%	41.9%
American Indian	0.0%	0.0%	4.7%	0.0%	23.4%
Alaska Native	0.0%	0.0%	0.1%	0.0%	0.2%
Native Hawaiian	0.0%	0.0%	0.2%	0.0%	1.4%
Guamanian	0.0%	0.0%	0.0%	0.0%	0.2%
Samoan	0.0%	0.0%	0.0%	0.0%	0.9%
Pacific Islander	0.0%	0.0%	0.3%	0.0%	2.6%
Asian Indian	0.0%	0.0%	0.2%	21.0%	4.4%
Filipino	0.0%	0.0%	0.7%	34.1%	8.1%
Chinese	0.0%	0.0%	1.5%	17.1%	7.1%
Japanese	0.0%	0.0%	0.2%	6.0%	5.1%
Korean	0.0%	0.0%	0.3%	10.3%	5.3%
Vietnamese	0.0%	0.0%	0.2%	10.3%	2.6%
Other Asian	0.0%	0.0%	0.4%	13.5%	3.7%
Other	0.0%	0.0%	27.2%	0.0%	11.5%
Refuse	0.0%	0.0%	1.2%	0.0%	2.5%
Don't Know	0.0%	0.0%	3.7%	0.0%	1.2%
Hispanic	0.0%	0.0%	100.0%	0.0%	0.0%

Table 18: Granular Race/Ethnicity Data by Condition

	F		QC1		C		0.5		0.4		0.3		QC2		0.2		0.1	
	n	%	n	%	n	%	n	%	n	%	n	%	n	%	n	%	n	%
White	8,804	74	8,461	75	7,320	76	7,127	76	7,083	76	6,977	77	7,300	76	6,557	77	4,211	80
Black	2,518	21	2,375	21	1,918	20	1,840	20	1,825	20	1,785	20	1,903	20	1,631	19	870	17
American Indian	406	3.4	387	3.4	326	3.4	314	3.4	310	3.4	303	3.3	320	3.3	289	3.4	182	3.5
Alaska Native	5	0.04	3	0.03	2	0.02	1	0.01	1	0.01	1	0.01	2	0.02	1	0.01	1	0.02
Native Hawaiian	23	0.19	22	0.19	20	0.21	20	0.21	20	0.22	20	0.22	19	0.2	18	0.21	6	0.11
Guamanian	2	0.02	2	0.02	2	0.02	2	0.02	2	0.02	2	0.02	0	0	2	0.02	1	0.02
Samoan	12	0.1	12	0.11	11	0.11	11	0.12	11	0.12	11	0.12	12	0.12	11	0.13	7	0.13
Pacific Islander	40	0.34	36	0.32	31	0.32	30	0.32	30	0.32	30	0.33	30	0.31	27	0.32	17	0.32
Asian Indian	114	0.96	108	0.95	92	0.96	88	0.94	88	0.95	86	0.95	90	0.93	79	0.93	50	0.95
Filipino	203	1.7	191	1.7	152	1.6	150	1.6	149	1.6	146	1.6	158	1.6	135	1.6	95	1.8
Chinese	167	1.4	157	1.4	124	1.3	121	1.3	121	1.3	119	1.3	135	1.4	116	1.4	63	1.2
Japanese	84	0.71	82	0.72	60	0.62	59	0.63	59	0.64	57	0.63	72	0.75	54	0.63	41	0.78
Korean	100	0.84	98	0.86	80	0.83	78	0.84	78	0.84	76	0.84	85	0.88	70	0.82	48	0.91
Vietnamese	63	0.53	60	0.53	47	0.49	45	0.48	44	0.48	43	0.47	50	0.52	42	0.49	28	0.53
Other Asian	90	0.76	86	0.76	74	0.77	69	0.74	69	0.74	67	0.74	69	0.72	62	0.73	39	0.74
Other	800	6.7	761	6.7	581	6	567	6.1	561	6.1	549	6	645	6.7	513	6	292	5.6
Refuse	59	0.5	56	0.49	41	0.43	39	0.42	39	0.42	37	0.41	44	0.46	33	0.39	16	0.3
Don't Know	104	0.88	97	0.85	77	0.8	75	0.8	75	0.81	74	0.81	84	0.87	72	0.85	38	0.72
Hispanic	2,411	21	2,312	21	1,845	19	1,799	20	1,788	20	1,746	19	1,966	21	1,630	19	960	18

Table 19: Granular Race/Ethnicity Variables: Bivariate Models, Pt. 1

	T	C	0.5	0.4	0.3	R	0.2	0.1
	OR<br>[90% CI]<br>p	OR<br>[90% CI]<br>p	OR<br>[90% CI]<br>p	OR<br>[90% CI]<br>p	OR<br>[90% CI]<br>p	OR<br>[90% CI]<br>p	OR<br>[90% CI]<br>p	OR<br>[90% CI]<br>p
White	.66***<br>.55-.80<br><.001	.58***<br>.53-.64<br><.001	.59***<br>.53-.65<br><.001	.59***<br>.54-.65<br><.001	.58***<br>.53-.64<br><.001	.64***<br>.58-.71<br><.001	.60***<br>.55-.65<br><.001	.56***<br>.51-.61<br><.001
Black	1.43***<br>1.17-1.74<br><.001	1.43***<br>1.29-1.59<br><.001	1.47***<br>1.33-1.62<br><.001	1.47***<br>1.33-1.63<br><.001	1.47***<br>1.33-1.62<br><.001	1.53***<br>1.37-1.70<br><.001	1.51***<br>1.37-1.65<br><.001	1.67***<br>1.52-1.83<br><.001
American Indian	1.07<br>.65-1.67<br>.769	1.04<br>.80-1.32<br>.779	1.07<br>.84-1.35<br>.570	1.10<br>.87-1.38<br>.419	1.12<br>.89-1.40<br>.338	1.16<br>.90-1.47<br>.240	1.02<br>.82-1.27<br>.838	.97<br>.80-1.19<br>.798
Alaska Native	14.58**<br>1.92-88.19<br>.003	6.33*<br>1.05-48.11<br>.043	14.61*<br>2.16-285.80<br>.016	14.19*<br>2.10-277.71<br>.018	13.12*<br>1.94-256.66<br>.021	6.43*<br>1.06-48.83<br>.042	10.11*<br>1.50-197.83<br>.039	3.17<br>.47-62.03<br>.302
Native Hawaiian	.99<br>.06-4.73<br>.993	.63<br>.15-1.85<br>.459	.55<br>.13-1.60<br>.329	.53<br>.13-1.55<br>.307	.49<br>.12-1.43<br>.250	.90<br>.26-2.40<br>.850	.70<br>.23-1.76<br>.483	2.25<br>.93-6.24<br>.088
Guamanian	.00<br>NA-3.46e+11<br>.967	.00<br>NA-125672.14<br>.942	.00<br>NA-108642.02<br>.941	.00<br>NA-105570.45<br>.941	.00<br>NA-97579.29<br>.941	451815.50<br>.00-NA<br>.926	.00<br>NA-75234.92<br>.939	.79<br>.03-20.04<br>.869

Table 20: Granular Race/Ethnicity Variables: Bivariate Models, Pt. 2

	T	C	0.5	0.4	0.3	R	0.2	0.1
	OR<br>[90% CI]<br>p	OR<br>[90% CI]<br>p	OR<br>[90% CI]<br>p	OR<br>[90% CI]<br>p	OR<br>[90% CI]<br>p	OR<br>[90% CI]<br>p	OR<br>[90% CI]<br>p	OR<br>[90% CI]<br>p
Samoan	.00<br>NA-835.53<br>.964	.38<br>.02-1.97<br>.358	.33<br>.02-1.70<br>.290	.32<br>.02-1.66<br>.278	.30<br>.02-1.53<br>.246	.00<br>NA-.34<br>.938	.23<br>.01-1.18<br>.158	.57<br>.17-1.77<br>.331
Pacific Islander	2.43<br>.73-6.10<br>.093	1.23<br>.55-2.47<br>.592	1.22<br>.56-2.41<br>.592	1.18<br>.55-2.34<br>.648	1.09<br>.51-2.16<br>.810	1.43<br>.66-2.83<br>.329	1.22<br>.61-2.32<br>.562	1.07<br>.57-2.04<br>.828
Asian Indian	1.21<br>.47-2.54<br>.647	1.01<br>.62-1.58<br>.971	1.08<br>.68-1.65<br>.737	1.05<br>.66-1.60<br>.837	1.07<br>.68-1.62<br>.767	1.14<br>.71-1.77<br>.563	1.12<br>.74-1.66<br>.579	1.01<br>.70-1.48<br>.940
Filipino	1.38<br>.72-2.38<br>.287	1.42*<br>1.02-1.95<br>.030	1.29<br>.94-1.76<br>.110	1.29<br>.93-1.75<br>.112	1.28<br>.94-1.74<br>.112	1.22<br>.87-1.69<br>.237	1.28<br>.95-1.71<br>.103	.90<br>.68-1.19<br>.454
Chinese	1.40<br>.69-2.53<br>.311	1.47*<br>1.03-2.07<br>.030	1.39<br>.98-1.95<br>.058	1.35<br>.95-1.89<br>.083	1.33<br>.94-1.85<br>.101	1.01<br>.68-1.48<br>.941	1.11<br>.79-1.54<br>.531	1.31<br>.96-1.81<br>.090
Japanese	.53<br>.09-1.68<br>.376	1.69*<br>1.03-2.69<br>.030	1.55<br>.95-2.45<br>.067	1.51<br>.93-2.38<br>.087	1.56<br>.97-2.44<br>.059	.71<br>.37-1.26<br>.277	1.41<br>.89-2.18<br>.136	.83<br>.54-1.28<br>.395

Table 21: Granular Race/Ethnicity Variables: Bivariate Models, Pt. 3

	T	C	0.5	0.4	0.3	R	0.2	0.1
	OR<br>[90% CI]<br>p	OR<br>[90% CI]<br>p	OR<br>[90% CI]<br>p	OR<br>[90% CI]<br>p	OR<br>[90% CI]<br>p	OR<br>[90% CI]<br>p	OR<br>[90% CI]<br>p	OR<br>[90% CI]<br>p
	.44	1.05	1.03	1.00	1.03	.75	1.08	.86
	.07-1.40	.63-1.69	.62-1.62	.61-1.58	.64-1.61	.42-1.27	.70-1.65	.58-1.27
Korean	.255	.831	.907	.998	.885	.314	.716	.444
	1.09	1.44	1.46	1.53	1.53	1.11	1.26	.99
	.27-2.95	.79-2.49	.82-2.48	.87-2.59	.88-2.56	.58-1.99	.73-2.11	.60-1.64
Vietnamese	.884	.210	.175	.120	.119	.730	.382	.970
	1.01	.91	1.11	1.08	1.13	1.31	1.14	1.04
	.31-2.44	.51-1.52	.66-1.78	.65-1.73	.69-1.78	.78-2.09	.72-1.77	.68-1.58
Other Asian	.979	.737	.675	.761	.627	.287	.563	.867
	1.13	1.65***	1.55***	1.56***	1.55***	1.03	1.45***	1.41***
	.79-1.55	1.40-1.94	1.32-1.81	1.33-1.83	1.32-1.81	.86-1.23	1.25-1.69	1.22-1.64
Other	.486	<.001	<.001	<.001	<.001	.744	<.001	<.001
	1.17	1.86*	1.88*	1.82*	1.95*	1.46	2.00**	2.14**
	.28-3.18	1.04-3.19	1.07-3.18	1.04-3.09	1.13-3.29	.79-2.57	1.18-3.34	1.23-3.91
Refuse	.793	.029	.023	.029	.013	.205	.009	.010

Table 22: Adjusted Models Output, as Pre-registered (Pt. 1). ***: p<.001; **: p<.01; *:p<.05

	0.5	0.4	0.3	0.2	0.1
Variable	OR [95% CI]	OR [95% CI]	OR [95% CI]	OR [95% CI]	OR [95% CI]
Intercept	.18*** [.15-.22]	.18*** [.16-.22]	.20*** [.17-.23]	.25*** [.21-.29]	.80** [.70-.92]
Sex (Male)	1.09 [.96-1.23]	1.11 [.99-1.26]	1.17** [1.04-1.32]	1.31*** [1.17-1.46]	1.42*** [1.29-1.57]
Household Income (ref: $100-$200k)					
$0-$25k	1.15 [.93-1.44]	1.17 [.94-1.45]	1.11 [.90-1.38]	1.13 [.93-1.38]	1.06 [.88-1.28]
$25-$50k	.89 [.73-1.09]	.91 [.75-1.11]	.92 [.76-1.12]	.87 [.73-1.04]	.89 [.76-1.05]
$50-$75k	.92 [.76-1.10]	.92 [.76-1.10]	.92 [.77-1.10]	.93 [.79-1.10]	1.09 [.94-1.26]
$75-$100k	.96 [.80-1.14]	.96 [.81-1.13]	.98 [.83-1.16]	.94 [.80-1.09]	.92 [.80-1.05]
>$200k	.98 [.81-1.18]	.98 [.82-1.18]	1.00 [.83-1.19]	.98 [.82-1.15]	.96 [.82-1.11]
Highest Parental Education (ref: College Degree)					
<High School	1.49** [1.10-2.00]	1.50** [1.11-2.01]	1.44* [1.07-1.93]	1.49** [1.12-1.97]	1.35* [1.02-1.79]
HS Grad.	1.25 [.99-1.58]	1.24 [.99-1.56]	1.26* [1.01-1.58]	1.29* [1.04-1.59]	1.23* [1.01-1.50]
Some College	1.02 [.86-1.20]	1.03 [.87-1.21]	1.03 [.88-1.20]	1.10 [.95-1.27]	1.03 [.91-1.18]
Graduate	1.13 [.98-1.30]	1.12 [.98-1.29]	1.12 [.98-1.29]	1.14 [1.00-1.29]	1.04 [.93-1.17]
Census Race/Ethnicity (ref: White)					
Black	1.69*** [1.40-2.04]	1.63*** [1.35-1.97]	1.59*** [1.32-1.91]	1.53*** [1.29-1.82]	1.48*** [1.26-1.75]
Hispanic	1.20* [1.02-1.40]	1.19* [1.02-1.39]	1.21* [1.04-1.40]	1.14 [.99-1.32]	1.16* [1.02-1.32]
Asian	1.92*** [1.38-2.64]	1.88*** [1.35-2.58]	2.05*** [1.48-2.80]	1.96*** [1.44-2.66]	1.49** [1.10-2.01]
Other	1.34*** [1.13-1.59]	1.34*** [1.13-1.59]	1.28** [1.08-1.52]	1.18* [1.00-1.38]	1.12 [.97-1.29]

Table 23: Adjusted Models Output, as Pre-registered (Pt. 2). ***: p<.001; **: p<.01; *:p<.05

	0.5	0.4	0.3	0.2	0.1
Variable	OR [95% CI]	OR [95% CI]	OR [95% CI]	OR [95% CI]	OR [95% CI]
KSADS Trauma Count (ref: 0 Exposures)					
1 Trauma	1.06 [.93-1.19]	1.05 [.93-1.19]	1.05 [.93-1.18]	1.03 [.92-1.15]	.96 [.87-1.06]
>=2 Trauma	1.12 [.93-1.34]	1.08 [.90-1.29]	1.06 [.89-1.26]	1.13 [.96-1.33]	1.20* [1.03-1.40]
Pubertal Status (ref: pre-pubertal)					
Early Puberty	1.02 [.90-1.17]	1.03 [.90-1.17]	1.01 [.89-1.15]	1.07 [.95-1.20]	1.07 [.96-1.19]
Mid Puberty	1.05 [.90-1.24]	1.06 [.90-1.24]	1.07 [.91-1.24]	1.08 [.94-1.25]	1.25*** [1.10-1.42]
Late Puberty	1.73** [1.16-2.55]	1.71** [1.15-2.52]	1.61* [1.08-2.38]	1.63* [1.11-2.37]	1.31 [.91-1.88]
Post Pubertal	.47 [.02-2.73]	.47 [.02-2.70]	.45 [.02-2.59]	1.55 [.31-6.50]	1.37 [.33-6.82]
Area Disadvantage	.70*** [.64-.77]	.72*** [.66-.78]	.73*** [.67-.79]	.76*** [.71-.83]	.86*** [.80-.93]
Child Opportunity	.78*** [.71-.86]	.80*** [.73-.88]	.80*** [.73-.87]	.82*** [.75-.90]	.84*** [.78-.92]
NIHTB Flanker	1.02 [.95-1.09]	1.01 [.95-1.09]	1.03 [.96-1.10]	1.02 [.96-1.09]	1.01 [.96-1.07]
NIHTB Crystalized	1.06 [.94-1.18]	1.05 [.94-1.17]	1.08 [.97-1.21]	1.09 [.99-1.21]	1.16** [1.06-1.27]
NIHTB Total	.88 [.77-1.00]	.88 [.78-1.00]	.84* [.74-.96]	.84** [.74-.94]	.81*** [.73-.90]
WISC V Matrix	1.00 [.95-1.06]	.99 [.93-1.05]	.98 [.93-1.04]	.95 [.90-1.00]	.95* [.90-.99]
Psychopathology	1.11*** [1.05-1.17]	1.11*** [1.05-1.17]	1.11*** [1.06-1.17]	1.13*** [1.07-1.18]	1.11*** [1.06-1.16]
Internalizing	.98 [.92-1.03]	.97 [.92-1.02]	.96 [.91-1.01]	.95 [.91-1.00]	.92*** [.88-.96]
Externalizing	.94* [.89-.99]	.94* [.89-.99]	.94* [.90-.99]	.94* [.89-.99]	.94** [.90-.98]
Age	.85*** [.81-.90]	.84*** [.80-.89]	.83*** [.79-.88]	.82*** [.78-.86]	.81*** [.78-.85]

Burley, D. T., Anning, K. L., & van Goozen, S. H. M. (2022). The association between hyperactive behaviour and cognitive inhibition impairments in young children. *Child Neuropsychology, 28*(3), 302–317. doi:10.1080/09297049.2021.1976128

Canivez, G. L., McGill, R. J., Dombrowski, S. C., Watkins, M. W., Pritchard, A. E., & Jacobson, L. A. (2020). Construct Validity of the WISC-V in Clinical Cases: Exploratory and Confirmatory Factor Analyses of the 10 Primary Subtests. *Assessment, 27*(2), 274–296. doi:10.1177/1073191118811609

Cheng, T. W., Magis-Weinberg, L., Guazzelli Williamson, V., Ladouceur, C. D., Whittle, S. L., Herting, M. M., Uban, K. A., Byrne, M. L., Barendse, M. E. A., Shirtcliff, E. A., & Pfeifer, J. H. (2021). A Researcher's Guide to the Measurement and Modeling of Puberty in the ABCD Study® at Baseline. *Frontiers in Endocrinology, 12.* doi:10.3389/fendo.2021.608575

Cosgrove, K. T., McDermott, T. J., White, E. J., Mosconi, M. W., Thompson, W. K., Paulus, M. P., Cardenas-Iniguez, C., & Aupperle, R. L. (2022). Limits to the generalizability of resting-state functional magnetic resonance imaging studies of youth: An examination of ABCD Study® baseline data. *Brain Imaging and Behavior, 16*(4), 1919–1925. doi:10.1007/s11682-022-00665-2

Clark, D. A., Hicks, B. M., Angstadt, M., Rutherford, S., Taxali, A., Hyde, L., Weigard, A., Heitzeg, M. M., & Sripada, C. (2021). The General Factor of Psychopathology in the Adolescent Brain Cognitive Development (ABCD) Study: A Comparison of Alternative Modeling Approaches. In *Clinical psychological science: A journal of the Association for Psychological Science, 9*(2), 169–182. doi:10.1177/2167702620959317

Evans, G. W., & Kim, P. (2010). Multiple risk exposure as a potential explanatory mechanism for the socioeconomic status-health gradient: Multiple risk exposure and SES-health gradient. *Annals of the New York Academy of Sciences, 1186*(1), 174–189. doi:10.1111/j.1749-6632.2009.05336.x

Fadus, M. C., Valadez, E. A., Bryant, B. E., Garcia, A. M., Neelon, B., Tomko, R. L., & Squeglia, L. M. (2021). Racial Disparities in Elementary School Disciplinary Actions: Findings From the ABCD Study. *Journal of the American Academy of Child & Adolescent Psychiatry, 60*(8), 998–1009. doi:10.1016/j.jaac.2020.11.017

Fan, C. C., Marshall, A., Smolker, H., Gonzalez, M. R., Tapert, S. F., Barch, D. M., Sowell, E., Dowling, G. J., Cardenas-Iniguez, C., Ross, J., Thompson, W. K., & Herting, M. M. (2021). Adolescent Brain Cognitive Development (ABCD) study Linked External Data (LED): Protocol and practices for geocoding and assignment of environmental data. *Developmental Cognitive Neuroscience, 52*, 101030. doi:10.1016/j.dcn.2021.101030

Heeringa, S., & Berglund, P. (2020). A Guide for Population-based Analysis of the Adolescent Brain Cognitive Development (ABCD) Study Baseline Data. doi:10.1101/2020.02.10.942011

McLaughlin, K. A., Koenen, K. C., Hill, E. D., Petukhova, M., Sampson, N. A., Zaslavsky, A. M., & Kessler, R. C. (2013). Trauma exposure and Posttraumatic Stress Disorder in a national sample of adolescents. *Journal of the American Academy of Child and Adolescent Psychiatry, 52*(8), 815-830.e14. doi:10.1016/j.jaac.2013.05.011

Petersen, A. C., Crockett, L., Richards, M., & Boxer, A. (1988). A self-report measure of pubertal status: Reliability, validity, and initial norms. *Journal of Youth and Adolescence, 17*(2), 117–133. doi:10.1007/BF01537962

Rakesh, D., Zalesky, A., & Whittle, S. (2021). Similar but distinct – Effects of different socioeconomic indicators on resting state functional connectivity: Findings from the Adolescent Brain Cognitive Development (ABCD) Study®. *Developmental Cognitive Neuroscience, 51*, 101005. doi:10.1016/j.dcn.2021.101005

Saragosa-Harris, N. M., Chaku, N., MacSweeney, N., Guazzelli Williamson, V., Scheuplein, M., Feola, B., Cardenas-Iniguez, C., Demir-Lira, E., McNeilly, E. A., Huffman, L. G., Whitmore, L., Michalska, K. J., Damme, K. S., Rakesh, D., & Mills, K. L. (2022). A practical guide for researchers and reviewers using the ABCD Study and other large longitudinal datasets. *Developmental Cognitive Neuroscience, 55*, 101115. doi:10.1016/j.dcn.2022.101115

Singh, G. K. (2003). Area Deprivation and Widening Inequalities in US Mortality, 1969–1998. *American Journal of Public Health, 93*(7), 1137–1143. doi:10.2105/AJPH.93.7.1137

Thompson, W. K., Barch, D. M., Bjork, J. M., Gonzalez, R., Nagel, B. J., Nixon, S. J., & Luciana, M. (2019). The structure of cognition in 9 and 10 year-old children and associations with problem behaviors: Findings from the ABCD study's baseline neurocognitive battery. *Developmental Cognitive Neuroscience, 36*, 100606. doi:10.1016/j.dcn.2018.12.004

Townsend, L., Kobak, K., Kearney, C., Milham, M., Andreotti, C., Escalera, J., Alexander, L., Gill, M. K., Birmaher, B., Sylvester, R., Rice, D., Deep, A., & Kaufman, J. (2020). Development of Three Web-Based Computerized Versions of the Kiddie Schedule for Affective Disorders and Schizophrenia Child Psychiatric Diagnostic Interview: Preliminary Validity Data. *Journal of the American Academy of Child & Adolescent Psychiatry, 59*(2), 309–325. doi:10.1016/j.jaac.2019.05.009

6 SessionInfo

This output describes the environment in which the statistical code was run including package versions.

R version 4.4.2 (2024-10-31)

Platform: x86_64-pc-linux-gnu

locale: *LC_CTYPE=en_US.UTF-8*, *LC_NUMERIC=C*, *LC_TIME=en_US.UTF-8*, *LC_COLLATE=en_US.UTF-8*, *LC_MONETARY=en_US.UTF-8*, *LC_MESSAGES=en_US.UTF-8*, *LC_PAPER=en_US.UTF-8*, *LC_NAME=C*, *LC_ADDRESS=C*, *LC_TELEPHONE=C*, *LC_MEASUREMENT=en_US.UTF-8* and *LC_IDENTIFICATION=C*

attached base packages: *stats*, *graphics*, *grDevices*, *utils*, *datasets*, *methods* and *base*

other attached packages: *emmeans(v.1.10.4)*, *lmerTest(v.3.1-3)*, *lme4(v.1.1-35.5)*, *Matrix(v.1.7-0)*, *ggupset(v.0.4.0)*, *openxlsx(v.4.2.5.2)*, *patchwork(v.1.3.0)*, *viridis(v.0.6.5)*, *viridisLite(v.0.4.2)*, *huxtable(v.5.5.6)*, *hexbin(v.1.28.3)*, *pander(v.0.6.5)*, *ggExtra(v.0.10.1)*, *ggthemes(v.5.1.0)*, *lubridate(v.1.9.3)*, *forcats(v.1.0.0)*, *stringr(v.1.5.1)*, *dplyr(v.1.1.4)*, *purrr(v.1.0.2)*, *readr(v.2.1.5)*, *tidyr(v.1.3.1)*, *tibble(v.3.2.1)*, *ggplot2(v.3.5.1)*, *tidyverse(v.2.0.0)* and *rmarkdown(v.2.27)*

loaded via a namespace (and not attached): *Exact(v.3.2)*, *tidyselect(v.1.2.1)*, *rootSolve(v.1.8.2.4)*, *farver(v.2.1.2)*, *fastmap(v.1.2.0)*, *bayestestR(v.0.15.0)*, *promises(v.1.3.0)*, *digest(v.0.6.36)*, *timechange(v.0.3.0)*, *estimability(v.1.5.1)*, *mime(v.0.12)*, *lifecycle(v.1.0.4)*, *lmom(v.3.0)*, *magrittr(v.2.0.3)*, *compiler(v.4.4.2)*, *rlang(v.1.1.4)*, *tools(v.4.4.2)*, *utf8(v.1.2.4)*, *yaml(v.2.3.9)*, *data.table(v.1.16.0)*, *knitr(v.1.48)*, *expm(v.0.999-9)*, *miniUI(v.0.1.1.1)*, *withr(v.3.0.2)*, *numDeriv(v.2016.8-1.1)*, *datawizard(v.0.13.0)*, *grid(v.4.4.2)*, *fansi(v.1.0.6)*, *xtable(v.1.8-4)*, *e1071(v.1.7-14)*, *colorspace(v.2.1-0)*, *extrafontdb(v.1.0)*, *scales(v.1.3.0)*, *MASS(v.7.3-61)*, *insight(v.0.20.5)*, *cli(v.3.6.3)*, *mvtnorm(v.1.2-5)*, *crayon(v.1.5.3)*, *generics(v.0.1.3)*, *performance(v.0.12.3)*, *rstudioapi(v.0.16.0)*, *httr(v.1.4.7)*, *tzdb(v.0.4.0)*, *parameters(v.0.23.0)*, *readxl(v.1.4.3)*, *gld(v.2.6.6)*, *proxy(v.0.4-27)*, *minqa(v.1.2.7)*, *sdlabFunctions(v.0.2.0)*, *splines(v.4.4.2)*, *assertthat(v.0.2.1)*, *cellranger(v.1.1.0)*, *vctrs(v.0.6.5)*, *boot(v.1.3-30)*, *hms(v.1.1.3)*, *glue(v.1.8.0)*, *nloptr(v.2.1.1)*, *stringi(v.1.8.4)*, *gtable(v.0.3.5)*, *later(v.1.3.2)*, *extrafont(v.0.19)*, *munsell(v.0.5.1)*, *pillar(v.1.9.0)*, *htmltools(v.0.5.8.1)*, *R6(v.2.5.1)*, *evaluate(v.0.24.0)*, *shiny(v.1.8.1.1)*, *lattice(v.0.22-6)*, *httpuv(v.1.6.15)*, *class(v.7.3-22)*, *DescTools(v.0.99.56)*, *Rcpp(v.1.0.13)*, *zip(v.2.3.1)*, *coda(v.0.19-4.1)*, *gridExtra(v.2.3)*, *nlme(v.3.1-166)*, *Rttf2pt1(v.1.3.12)*, *xfun(v.0.45)* and *pkgconfig(v.2.0.3)*
